# Nanocellulose: Recent Fundamental Advances and Emerging Biological and Biomimicking Applications

**DOI:** 10.1002/adma.202004349

**Published:** 2020-12-02

**Authors:** Katja Heise, Eero Kontturi, Yagut Allahverdiyeva, Tekla Tammelin, Markus B. Linder, Nonappa, Olli Ikkala

**Affiliations:** ^1^ Department of Bioproducts and Biosystems Aalto University Espoo FI‐00076 Finland; ^2^ Center of Excellence in Molecular Engineering of Biosynthetic Hybrid Materials Research Aalto University FI‐00076 Finland; ^3^ Molecular Plant Biology Department of Biochemistry University of Turku Turku FI‐20014 Finland; ^4^ VTT Technical Research Centre of Finland Ltd VTT, PO Box 1000 FIN‐02044 Espoo Finland; ^5^ Department of Applied Physics Aalto University Espoo FI‐00076 Finland; ^6^ Faculty of Engineering and Natural Sciences Tampere University P.O. Box 541 Tampere FI‐33101 Finland

**Keywords:** cellulose nanocrystals, cellulose nanofibers, functional matter, nanocellulose, nanofibrillated cellulose

## Abstract

In the effort toward sustainable advanced functional materials, nanocelluloses have attracted extensive recent attention. Nanocelluloses range from rod‐like highly crystalline cellulose nanocrystals to longer and more entangled cellulose nanofibers, earlier denoted also as microfibrillated celluloses and bacterial cellulose. In recent years, they have spurred research toward a wide range of applications, ranging from nanocomposites, viscosity modifiers, films, barrier layers, fibers, structural color, gels, aerogels and foams, and energy applications, until filtering membranes, to name a few. Still, nanocelluloses continue to show surprisingly high challenges to master their interactions and tailorability to allow well‐controlled assemblies for functional materials. Rather than trying to review the already extensive nanocellulose literature at large, here selected aspects of the recent progress are the focus. Water interactions, which are central for processing for the functional properties, are discussed first. Then advanced hybrid gels toward (multi)stimuli responses, shape‐memory materials, self‐healing, adhesion and gluing, biological scaffolding, and forensic applications are discussed. Finally, composite fibers are discussed, as well as nanocellulose as a strategy for improvement of photosynthesis‐based chemicals production. In summary, selected perspectives toward new directions for sustainable high‐tech functional materials science based on nanocelluloses are described.

## Graphical abstract

Nanocelluloses have recently become the focal point of sustainable nanoconstruction. Beyond the classic approaches dealing with, for example, reinforced nanocomposites, barriers, films, and structural color, the fundamental interactions within nanocelluloses, multifunctional hybrid gels, multicomponent fibers, and nanocelluloses in artificial photosynthesis are reviewed.

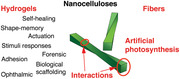

## 1 Introduction

Cellulose is the most widespread sustainable and renewable biopolymer on earth. In all green plants, the polymer chains of β‐(1‐4)‐linked‐d‐glucose repeating units assemble into nanosized, thread‐like agglomerates called microfibrils, interacting with hydrogen bonding and van der Waals interactions resulting in the structural scaffold of the fiber cell walls.^[^ ^1^ ^]^ Wood/plant‐based cellulose fibers have been used for centuries in materials such as papers and textiles. At the molecular level, various derivatives of cellulose chains have been used as binders, in food packaging, emulsifiers, and biomedical applications.^[^ ^2^ ^]^ More recently, the sustainable origin of cellulose has launched increasing efforts for its valorization in novel polymer technologies. Partly because of such a tremendous industrial appeal of cellulose‐based materials, the fundamental aspects of cellulose research have traditionally been overshadowed by the engineering approaches. While the bulk of the investigations still remains application‐driven, the past two decades have also demonstrated a significant rise in the efforts to understand the fundamental concepts of various celluloses. For example, the native crystalline structure, heterogeneous chemical modification, interactions between cellulose and water, and the precise morphology of the cellulose microfibrils and their assemblies are issues that have undergone substantial revisions during the present century.^[^ ^1^, ^3^ ^]^

Importantly, the 21st century has also witnessed the rise of nanocelluloses: a family of renewable high aspect ratio nanoparticles with high mechanical properties bearing chemical reactive groups on their surfaces, allowing functionalization, taken that the chemistry is properly mastered. Nanocelluloses comprise of crystalline rod‐like cellulose nanocrystals (CNCs), as well as longer and more entangled cellulose nanofibers (CNFs), occasionally also denoted as microfibrillated cellulose (MFC), and bacterial cellulose (BC).^[^ ^4^, ^5^, ^6^, ^7^, ^8^, ^9^, ^10^ ^]^ The literature related to nanocelluloses grows rapidly, where nanocellulose based nanocomposites, films and fibers, structural colors, barrier properties, emulsions, and viscosity tunings have been among the focal areas in science and technology, already reviewed extensively.^[^ ^7^, ^8^, ^9^, ^10^, ^11^, ^12^, ^13^, ^14^, ^15^, ^16^, ^17^, ^18^ ^]^ Moreover, in wood‐based bulk materials, new directions have been taken, for example, combining strength and toughness, as recently reviewed.^[^ ^16^ ^]^

Sooner than replicating the recent existing general reviews on nanocellulose,^[^ ^11^, ^17^, ^18^ ^]^ here we aim to focus on some specific emerging areas, particularly those ones that have benefited from the newly minted fundamental findings. First, we present how new advances in fundamental cellulose science can be weaved with nanocellulose technology to offer new directions in the coming decade. Taken the importance of gels, generic routes for (multi)functional properties can be achieved upon exploiting the functionalities of the constituent structural components. Here we will focus on such hybrid gels, allowing stimuli‐responsivity, shape‐memory effects, self‐healing, biological functions, such as tissue scaffolding, gluing, as well as fingerprint detection. Finally, nanocelluloses have turned interesting in emerging green electronics and energy applications, as already reviewed.^[^ ^19^ ^]^ Instead of dwelling on, e.g., solar cells or thermoelectric routes, here we will briefly focus on an emerging field of an artificial photosynthesis based on nanocelluloses. As a final remark, some of the concepts show multiple functionalities. To suppress repetitions, we may discuss them mainly in the context of one functionality, still pinpointing the other functionalities.

We emphasize the fundamental recent advances in this review. For example, truly novel insights to plant cell wall ultrastructure, size, and shape of the cellulose microfibril, and cellulose–water interactions have not been featured in material‐related reviews on nanocellulose, although they have been subject to specialized reviews.^[^ ^20^ ^]^ Likewise, the section on nanocellulose modification covers fresh ground by focusing on end‐wise modification of CNCs and novel (mainly <2 year old) accounts on polymer grafting on nanocellulose, thus distinguishing itself from the already well‐known nanocellulose‐based reviews,^[^ ^11^, ^17^ ^]^ or more specialized accounts without a broader materials perspective.^[^ ^21^, ^22^, ^23^ ^]^

## 2 Recent Progress in the Fundamental Understanding of Nanocellulose

**Figure 1:**
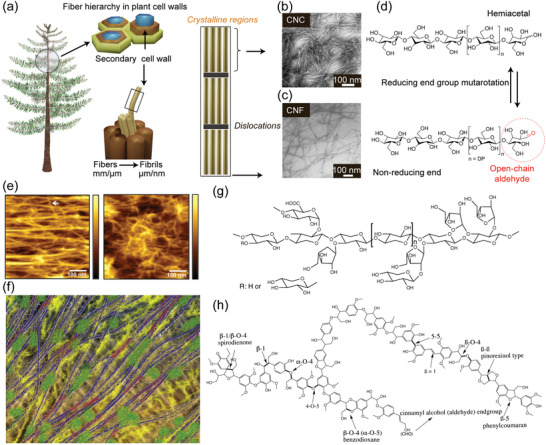
a) Schematic construction of the plant cell wall. b,c) TEM images of CNCs and CNFs, respectively. d) Chemical structures showing reducing end mutarotation. e) AFM images showing how primary wall consists of ordered microfibril layers. f) Schematic model for biomechanical hotspots (red): cellulose microfibrils in blue, pectins in yellow, and xyloglucan in green. g,h) Molecular structures of the main cell wall polymers, hemicellulose, and lignin structures, respectively. a,d) Adapted with permission.^[^ ^23^ ^]^ Copyright 2020, Wiley‐VCH. b) Adapted with permission.^[^ ^29^ ^]^ Copyright 2013, American Chemical Society. c) Adapted with permission.^[^ ^30^ ^]^ Copyright 2007, American Chemical Society. e) Reproduced with permission.^[^ ^28^ ^]^ Copyright 2020, American Chemical Society. f) Reproduced with permission.^[^ ^34^ ^]^ Copyright 2014, Elsevier Ltd.

The plant cell wall comprises semicrystalline cellulose microfibrils in an amorphous matrix of hemicellulose and lignin (**Figure** **1**). The challenges in structural characterization of the plant cell and its constituents can be categorized according to the length scales: micronscale concerning the cell wall layers, mesoscale concerning how the constituents are located with each other, nanoscale concerning the structure of the cellulose microfibril and its bundles, and molecular scale concerning the structure of the constituents.

### 2.1 Cell Wall Structure and the Location of Constituents

The crude overall view on the structure of the secondary and primary walls within the plant cell wall has been cemented for decades. The principal distinctions are within the constituent ratios as well as in cellulose microfibril orientation (Figure 1a).^[^ ^24^ ^]^ Recent research has shed light on the structure of the primary wall, which has—in a simplified representation—been considered to consist of a nonaligned microfibril network, resisting the turgor (osmotic) pressure while extending during the cell growth.^[^ ^24^ ^]^ For long, microscopic findings have suggested that the microfibrils do not just form an isotropic mesh in the primary wall.^[^ ^25^ ^]^ Recent studies have drawn a more accurate picture of a crossed polylamellate structure: aligned microfibrils form thin, yet continuous and discrete layers (lamellae) whose orientations change when progressing through the primary wall in the transverse direction.^[^ ^26^ ^]^ The shift in the microfibril orientation between the layers appeared to be random rather than gradual.^[^ ^26^, ^27^, ^28^ ^]^

Another new development in understanding the primary cell wall structure concerns the interplay of noncellulosic polysaccharides and cellulose with each other. Notably, a hemicellulose called xyloglucan has long been thought to strengthen the primary wall by noncovalently linking the load‐bearing cellulose microfibrils by extended chains.^[^ ^25^, ^26^, ^27^, ^28^, ^29^, ^30^, ^31^ ^]^ This “tethered network model” was cast in doubt based on the studies with xyloglucan deficient mutant *Arabidopsis* plants.^[^ ^32^, ^33^ ^]^ According to an alternative model, cellulose microfibrils are directly in contact with one another in “biomechanical hotspots” while a small amount of coiled (rather than extended) xyloglucan is acting as an adhesive in some cases (Figure 1d).^[^ ^33^, ^34^ ^]^ Meanwhile, the role of pectins, acidic polysaccharides found in the primary wall, is still unclear,^[^ ^35^ ^]^ as are several other issues on the functions and locations of noncellulosic polysaccharides in the primary wall.^[^ ^36^ ^]^

**Figure 2:**
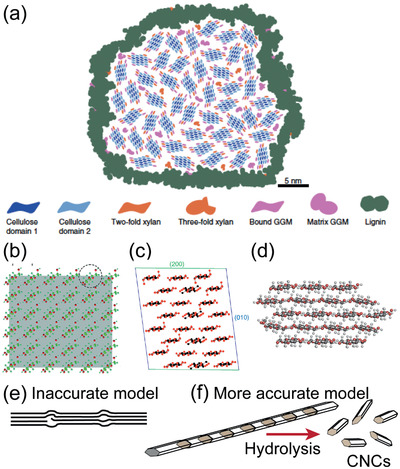
a) Schematic representation of a cellulose microfibril bundle in a softwood cell wall: the bundle is surrounded by lignin, and individual microfibrils are loosely linked by amorphous xylan and GGM while some xylan and GGM are tightly bound on the microfibrils. b) The much debated 6 × 6 chain model of cellulose. c,d) It is currently ousted in favor of the 6 × 4 model (c) or, even more likely, the 18‐chain model (34443 form) (d). e) Fringed‐fibrillar model of cellulose microfibril, more accurately nowadays presented by a semicrystalline microfibril where disordered regions are short defects rather than bulky amorphous regions. f) Nevertheless, the defects enable selective acid hydrolysis, leading to CNCs. a) Reproduced under the terms of the CC‐BY Creative Commons Attribution 4.0 International license (https://creativecommons.org/licenses/by/4.0/).^[^ ^38^ ^]^ Copyright 2019, The Authors, published by Springer Nature. b) Reproduced with permission.^[^ ^39^ ^]^ Copyright 2010, American Chemical Society. c) Reproduced with permission.^[^ ^40^ ^]^ Copyright 2011, National Academy of Sciences, USA. d) Reproduced under the terms of the CC‐BY Creative Commons Attribution 4.0 International license (https://creativecommons.org/licenses/by/4.0/).^[^ ^41^ ^]^ Copyright 2018, The Authors, published by Springer Nature. f) Reproduced with permission.^[^ ^42^ ^]^ Copyright 2016, Wiley‐VCH.

Advances in genome sequencing and identification of mutants particularly in *Arabidopsis* have also enabled a more detailed understanding of the secondary wall, whose structure most modern and historical accounts often simplify as having aligned microfibrils embedded in an amorphous lignin–hemicellulose matrix or similar. Multidimensional NMR studies,^[^ ^37^ ^]^ on cellulose deficient mutants have suggested that hemicellulose xylan adapts twofold screw conformations, analogous to the cellulose I crystal, on a cellulose microfibril. A follow‐up study,^[^ ^38^ ^]^ on a spruce (*Picea abies*) secondary wall found that also hemicellulose galactoglucomannan (GGM) can be found in a two‐fold screw conformation, thereby intimately binding with cellulose microfibrils. **Figure** **2**a shows elaborate schematics of a cellulose macrofibril, i.e., a bundle of microfibrils. In addition to the matrix amorphous form—in a threefold screw conformation—GGM and xylan can bind directly on the same microfibril. Occasionally lignin surrounding the macrofibril is found in close proximity of the hemicellulose, probably provoked by covalent bonds between the two.

### 2.2 Cellulose Microfibrils

Despite the considerable leap in analyzing the unit cell of native cellulose I_α_ and I_β_ crystals earlier this century,^[^ ^43^, ^44^ ^]^ the morphology of the native microfibril has been subject to constant debate, particularly during the past decade. It is generally accepted that the biological origin dictates the microfibril width and that the width becomes smaller as the evolutionary status of the plant is elevated. As a result, algae have the largest microfibrils (≈20 nm diameter), whereas trees possess the smallest ones (≈3–4 nm in diameter).^[^ ^45^ ^]^ These smallest microfibrils were thought to consist of 36 cellulose chains (Figure 2b),^[^ ^39^, ^46^ ^]^ based on circumstantial evidence from the structure of the cellulose synthase enzyme.^[^ ^47^, ^48^ ^]^ The group of Michael Jarvis published a seminal paper in 2011 that questioned the 36 chain model, and suggested a 24 chain structure instead (Figure 2c), based on an elaborate study with FTIR, NMR, and diffraction.^[^ ^40^ ^]^ Molecular dynamics (MD) simulations triggered researchers to further reduce the number of chains to 18,^[^ ^49^ ^]^ and this has stuck as the staple of the smallest microfibril within the past five years.^[^ ^38^, ^41^, ^50^ ^]^ Kubicki et al.^[^ ^41^ ^]^ explored a number of different shapes and concluded that the 34443 model (Figure 2d) is the most probable for the 18 chain model. The high amount of surfaces in the small 18 chain model explains why modifications of the microfibril surface already have a significant impact on the mass density of the microfibril. For example, oxidizing the primary hydroxyl (OH) groups on the microfibril surface via TEMPO‐oxidation changes the mass density from 1.60 to 1.70 g cm^−3^, simply due to an increase of molecular weight on surface anhydroglucose units.^[^ ^51^ ^]^

Semicrystallinity of a cellulose microfibril is another distinct feature that has been revised during the 21st century. Traditionally, the cellulose microfibril has been thought to consist of crystalline domains that are interrupted by “amorphous domains” along the length of the microfibril. However, a neutron scattering study suggested that these domains are not actually bulky amorphous regions but are in fact very short (1–2 nm) and should, therefore, be called disordered domains or defects instead (Figure 2e).^[^ ^52^ ^]^ The short length of domains is probably the reason why the disordered domains are not visible even in high resolution microscopy and can be indirectly observed only as kinks,^[^ ^53^ ^]^ in the isolated CNFs. Consequently, the fact that quantifications of the crystallinity index usually give a crystallinity of ≈50–80% for native cellulose does not imply that 20–50% of cellulose is amorphous. Instead, the results are likely artifacts and the “true” amount of disordered cellulose is in the order of 1–3%.

Finally, the chirality of crystalline cellulose in a microfibril has been subject to renewed interest within recent years. MD simulations have suggested a chiral right‐handed twist in cellulose chains,^[^ ^54^ ^]^ and the native crystal.^[^ ^55^, ^56^ ^]^ Moreover, the chiral properties of many cellulosic materials—such as chiral nematic CNC suspensions,^[^ ^57^ ^]^ point toward chiral crystals but direct, quantitative experimental evidence has been difficult to gather until recently. Quantitative data for the twist was provided in two studies on the latter half of the past decade,^[^ ^53^, ^58^ ^]^ and further discussed in an electron microdiffraction study by Ogawa.^[^ ^59^ ^]^ Moreover, combined computational and experimental evidence was reported by Conley et al.^[^ ^60^ ^]^ Based on circular dichroism, the authors calculated a twist of 800 nm period for wood CNCs. CNCs are typically 50–300 nm in length and the length of the period could be the reason why the twist has been difficult to visualize. Right‐handed twist of CNC was also indirectly indicated by chiral plasmonics of gold nanoparticles adsorbed on the CNC surfaces.^[^ ^61^ ^]^ Furthermore, drying and processing of cellulose is bound to make twists more localized in the CNF.^[^ ^53^, ^62^, ^63^ ^]^

### 2.3 Isolation of Nanocellulose from the Cell Wall Matrix

The technical aspects of CNF,^[^ ^64^, ^65^ ^]^ and CNC,^[^ ^66^, ^67^ ^]^ isolation have been reviewed recently. Concerning CNFs, the predominant method of isolation has been the disintegration of the cell wall matrix through mechanical shear, ending up with the liberation of the microfibrils. Standard pretreatments have consisted of enzymatic digestion and TEMPO‐oxidation, where the latter is the only method that manages to individualize the microfibrils to CNFs efficiently. With wood‐based sources, this essentially means that TEMPO‐oxidized CNFs consist of long (5–10 µm) threads of highly monodisperse widths of 3–4 nm.^[^ ^68^ ^]^ Recently, phosphorylation,^[^ ^69^ ^]^ and periodate oxidation,^[^ ^70^ ^]^ have surfaced as suitable pretreatment techniques. All pretreatment methods are based on the same idea as TEMPO‐oxidation: addition of charge on the microfibril surface to facilitate the detachment of microfibrils from one another into CNFs. Curiously, the methods utilizing exclusively high shear result in more bent or curved CNFs than the approaches that rely on charge addition. Indeed, recent simulations have suggested that plastic deformations due to high shear may occur even in crystalline cellulose.^[^ ^71^ ^]^

The isolation of CNCs requires a chemical reaction to cut the cellulose chains from the disordered domains and liberate the rod‐like CNCs (Figure 2e). Traditionally, CNCs are prepared by treating fibers with 63–65% sulfuric acid, based on the seminal study of Mukherjee and Woods in 1953.^[^ ^72^ ^]^ However, this method amounts to poor yields (20–50%), high water consumption, and laborious purification procedures with centrifugation and dialysis.^[^ ^67^ ^]^ The discoveries of the longitudinal disorder of the microfibril (see the previous section and Figure 2e) imply that the theoretical yield of CNCs should be very high, in the order of >95% as the amount of disordered cellulose should be very low. Indeed, recent years have seen reports of novel technologies with appreciably high yields >80%.^[^ ^42^, ^73^, ^74^, ^75^ ^]^

### 2.4 Advances in Chemical Modification

**Figure 3:**
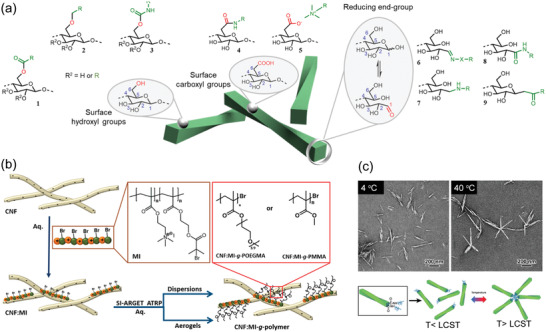
Chemical modification of nanocelluloses. a) Surface functionalities and common group‐specific derivatives: OH—esters (**1**), ethers (**2**), and carbamates (**3**); COOH—amides (**4**) and noncovalent complexes (**5**) (i.e., tertiary ammonium compounds); REG aldehyde (in equilibrium with α/β‐hemiacetal)—hydrazones (—N—NH—R) or oximes (—N—O—R) (**6**), amines (**7**), amides (**8**), and ketones (**9**). b) Polymer grafting‐from CNF modified with a cationic macroinitiator (MI) using SI‐ARGET ATRP in water. c) CNCs end‐functionalized with polyetheramines and TEM images showing the temperature‐induced formation of star‐shaped geometries above the lower‐critical solution temperature (LCST) of the polyetheramine chains. b) Reproduced with permission.^[^ ^109^ ^]^ Copyright 2019, American Chemical Society (https://pubs.acs.org/doi/10.1021/acs.biomac.9b00153; further permissions related to the material excerpted should be directed to the ACS). c) Reproduced with permission.^[^ ^99^ ^]^ Copyright 2019, American Chemical Society.

Chemical modification is the key to combine natural materials with the controllability of synthetic chemistry. The appeal behind tailoring the surface chemistry of nanocelluloses is far‐reaching, and the synthetic freedom provided by the multifunctional surface has been demonstrated in an immense variety of modification concepts, especially for CNCs.^[^ ^76^, ^77^, ^78^ ^]^ CNCs and CNF inherently bear surface hydroxyls and reducing end group (REG) aldehydes (**Figure** **3**a). As a polyalcohol, cellulose per se gives access to typical alcohol derivatives (Figure 3a, 1–3).^[^ ^76^, ^77^ ^]^ Nevertheless, nanocelluloses are far from behaving like normal alcohol which is, not least, a consequence of the heterogeneous reaction state when approaching their surfaces.^[^ ^76^ ^]^ Moreover, despite the high surface area, the overall chemical accessibility is governed by the colloidal stabilization of the nanoparticles in the reaction medium and their redispersibility, when provided in dry form. Additionally, the availability of reaction sites on the surface is often reduced throughout the nanocellulose isolation, i.e., during H_2_SO_4_ or H_3_PO_4_ hydrolysis, due to a partial surface substitution. Oxidative treatments (e.g., TEMPO‐oxidation,^[^ ^68^, ^73^, ^79^ ^]^ hydrolysis with dicarboxylic acids,^[^ ^80^, ^81^ ^]^ carboxymethylation^[^ ^82^, ^83^ ^]^) are an exception here. They introduce surface carboxyls, which facilitate covalent,^[^ ^84^, ^85^ ^]^ and noncovalent,^[^ ^86^, ^87^ ^]^ modification (**4**, **5** in Figure 3a). The directionality of native cellulose provides additional synthetic freedom: nanocelluloses bear aldehyde (hemiacetal) groups exclusively on one end,^[^ ^88^ ^]^ allowing topochemical modifications—a recently emerging trend in the realm of material concepts for CNCs.^[^ ^22^, ^23^ ^]^ Aldehyde‐specific modifications at the REG are, however, challenging since the availability of REG aldehydes is determined by the tautomeric equilibrium (mutarotation^[^ ^89^ ^]^) that favors the closed‐ring hemiacetals (Figure 3a). This equilibrium requires a precise control over the reaction conditions (i.e., catalysis, solvent nature, pH^[^ ^90^ ^]^) and a chemistry that catalyzes the ring‐opening to form stable glycoconjugates. Recent approaches toward CNC REG functionalization (Figure 3a) include hydrazine ligation (**6**),^[^ ^91^, ^92^, ^93^ ^]^ reductive amination (**7**),^[^ ^94^, ^95^, ^96^ ^]^ Pinnick oxidation followed by amide coupling (**8**),^[^ ^95^, ^97^, ^98^, ^99^ ^]^ and Knoevenagel condensation (**9**).^[^ ^100^ ^]^

The understanding of CNCs and CNFs as reaction templates and their integration into specific applications has resulted in tremendous synthetic advances, particularly in terms of macromolecular concepts (i.e., polymerization or ligation of biomolecules) and controllable systems. Polymer grafting initiated from the nanocellulose surface has been among the most trendsetting concepts in the last two decades. Especially, ring‐opening polymerizations (ROP) and controlled radical polymerizations (CRPs) have been tailored to the nanocellulose surface.^[^ ^21^, ^101^ ^]^ ROPs exploit surface hydroxyls or carboxyls as initiation sites for polymerizing cyclic monomers (e.g., ε‐caprolactone^[^ ^102^, ^103^ ^]^ or lactides^[^ ^104^, ^105^ ^]^). The chain propagation results in biodegradable polyester architectures, rendering ROPs particularly interesting in the biomedical realm. CRPs, on the other hand, have striking advantages in terms of precise control over grafting density, architecture, and polydispersity.^[^ ^21^ ^]^ A bottleneck, however, is the initial immobilization (e.g., via esterification) of a polymerization initiator on the nanocellulose surface.^[^ ^106^, ^107^ ^]^ Applied to CNCs and CNF, Cu‐catalyzed atom transfer radical polymerizations (ATRPs) are probably the most common CRPs due to their versatility.^[^ ^21^, ^101^ ^]^ Moreover, as an advanced generation of ATRPs, ARGET (activator regenerated by electron transfer) ATRP is less sensitive to oxygen and reduces the Cu‐catalyst level to ppm, rendering it a controllable technique for building polymer brushes from nanocelluloses (Figure 3b).^[^ ^108^, ^109^ ^]^ Applied to CNC REGs, surface‐initiated (SI) ATRP has been attempted for synthesizing Janus‐type colloids,^[^ ^97^, ^98^ ^]^ which may be the key to control the self‐assembly of CNCs into sophisticated 3D nanostructures. The crucial step here is the attachment of ATRP‐initiators selectively to the REGs. However, attempts reported so far resulted in patchy CNC‐polymer grafts, likely attributable to impurities on the CNC surface.^[^ ^97^, ^98^ ^]^ Lin et al., recently reported a successful end‐specific modification showing temperature‐triggered self‐assembly of Janus‐type CNCs, bearing polyetheramines grafted to the REGs, into star‐shaped geometries (Figure 3c).^[^ ^99^ ^]^ Overall, the possibility to come up with Janus‐type nanorod structures and similar anisotropic modifications with CNCs can potentially lead to conceptual advances in, e.g., responsive composite materials, interfacial engineering approaches, or templated assemblies. One genuine application of such structures could be in newly developed nanomachines.

The uniform or topochemical decoration of the nanocellulose surface, with active sites via grafting‐to methods is another approach to tailor nanocellulose‐based materials for specific applications. In this regard, especially click reactions, including Cu‐catalyzed azide–alkyne cycloadditions (CuAACs),^[^ ^110^, ^111^ ^]^ photoinduced thiol–ene or thiol–yne reactions,^[^ ^112^, ^113^, ^114^ ^]^ and the nitrile imine‐mediated tetrazole–ene cycloaddition (NITEC),^[^ ^115^, ^116^ ^]^ are a gateway toward an excellent atom‐economy, selectivity, and mild aqueous conditions. Moreover, the Cu‐free, light‐driven mechanisms of thiol‐click reactions and NITEC open‐up new avenues for the light‐based micropatterning of nanocellulose films and 3D structures.^[^ ^115^, ^117^, ^118^ ^]^

Indeed, the chemical pathways used to tailor nanocelluloses surfaces have entered the next level. Furthermore, recent developments have been significantly affected by the green chemistry concepts, which may, for instance, involve chemoenzymatic surface modifications of nanocelluloses.^[^ ^119^ ^]^ Besides synthetic aspects, advanced NMR techniques combined with the dissolution of the nanocellulose in an ionic liquid electrolyte, enable a precise analysis and quantification of the introduced functionalities, even at the REGs.^[^ ^100^, ^120^ ^]^ These are important conceptual advances as the quantification of surface modification has always been a severe bottleneck, particularly in the absence of elemental distinction.

### 2.5 Cellulose–Water Interactions

The relationship between cellulose‐based materials and water has been studied for roughly a century because of its immense practical implications.^[^ ^121^ ^]^ A piece of paper immediately gets soaked and easily disintegrates when immersed in water. By contrast, the water uptake by a piece of wood is relatively moderate because of hydrophobic lignin. In nanoscale, individual cellulose microfibrils do not swell in water—crystalline cellulose is impenetrable by water,^[^ ^122^, ^123^ ^]^—but it is the network of microfibrils that swells as the microfibril (and CNF) surfaces are highly hygroscopic. The amorphous hemicellulose structures add significantly to the water uptake because they swell more than the semicrystalline microfibrils.^[^ ^124^ ^]^

**Figure 4:**
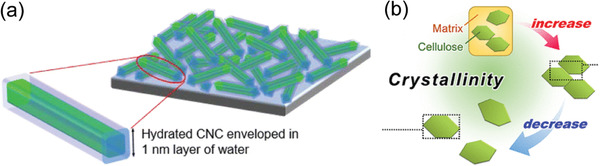
Schematic showing how CNCs in an ultrathin film are enveloped by 1 nm water layer throughout under high humidity. b) Schematic showing how an ordered stack (bundle) of microfibrils in a cell wall is disintegrated in CNF production, leading to a decrease in crystallinity. a) Reproduced with permission.^[^ ^130^ ^]^ Copyright 2015, American Chemical Society. b) Reproduced with permission.^[^ ^133^ ^]^ Copyright 2018, American Chemical Society (https://pubs.acs.org/doi/10.1021/acsanm.8b01438; further permissions related to the material excerpted should be directed to the ACS).

Exceeding the information retrieved by classical water retention and dynamic vapor sorption experiments, more sophisticated analytical approaches, such as NMR,^[^ ^125^ ^]^ neutron scattering,^[^ ^126^ ^]^ thermoporosimetry,^[^ ^124^, ^127^, ^128^ ^]^ and molecular dynamics simulations,^[^ ^126^, ^128^, ^129^ ^]^ have identified two species of water in cellulosic fibers, connected to the porous structure formed by microfibrils: i) tightly bound (nonfreezing) water directly associated with the crystalline cellulose surface and ii) weakly bound water between the microfibril network. Studies with CNC model films have shown that, at elevated humidity levels, roughly 1 nm water is deposited on crystalline cellulose (**Figure** **4**a).^[^ ^130^ ^]^ With CNF model films, in turn, distinct regions of swelling at 0–100%RH levels can be identified, partially affected by hemicellulose and the more flexible nature of CNFs in contrast to CNCs.^[^ ^131^ ^]^ Interestingly, a recent molecular dynamics study also highlighted the role of chirality of microfibrils in water uptake: disorder resulting from the twist allows water molecules to diffuse inside otherwise tight microfibril bundles.^[^ ^128^ ^]^

Removal of water, on the other hand, causes the pores to collapse and rewetting does not reversibly restore the swelling capability. This has traditionally been ascribed to irreversible microfibril aggregation, i.e., formation of microfibril bundles where the aggregation between individual fibrils is “tighter” or more “intimate” than within the bundles of the native cell wall (see, Figure 2a).^[^ ^132^ ^]^ CNFs and CNCs are usually prepared from highly processed cellulose where tight microfibril aggregation has often occurred. Most of the time, the isolation techniques attempt to disentangle the bundles, leading to as individualized fibrils or crystallites as possible. As mentioned, TEMPO‐oxidation manages to almost fully disassemble the microfibril bundles to individual CNFs,^[^ ^127^ ^]^ and this has recently been shown to result in decreased crystallinity after CNF isolation.^[^ ^133^ ^]^ The data suggest that the interfaces between the microfibrils in the original bundles are highly ordered and become disordered upon individualization of CNFs (Figure 4b). These findings lend credence to the hypothesis that a high degree of order, or even cocrystallization, emerges when elementary microfibrils associate by bundling within the cell wall.^[^ ^132^ ^]^ This association (or aggregation) occurs because of water removal between the microfibrils.

In terms of materials applications, the above account paints a familiar picture where cellulose–water interactions are viewed predominantly as a nuisance that is impairing the use of (nano)cellulose: water is detrimental to integrity and strength of a material, be it paper, cardboard, nanopaper, or composite. In this report, we want to highlight how the behavior with water can actually be seen as a benefit when building novel functional nanocellulose constructs. It is true that many commercial applications of nanocellulose already “excuse” the presence of water in, e.g., tissue growth or wound dressing applications that rely on nanocellulose hydrogels. So far, the utilization of highly specific water interactions with cellulose is at an early stage of the research, with only a handful of accounts in literature.

## 3 Nanocellulose‐Based Hybrid and Functional Hydrogels

There exists an extensive literature on how to achieve hydrogels based on cellulose nanofibers, cellulose nanocrystals, tunicates, and bacterial cellulose, as already reviewed.^[^ ^5^, ^134^, ^135^, ^136^, ^137^, ^138^, ^139^ ^]^ Beyond them, here the focus is on nanocellulosic hybrid hydrogels allowing stimulus‐responses, shape‐memory effects, self‐healing, actuation, electric properties, and biological functionalities, such as tissue scaffolding and ophthalmic applications, as well as fingerprint detection. Also wound healing is briefly discussed.^[^ ^140^, ^141^ ^]^ By hybrid nanocomposite nanocellulose gels, we mean that the composition involves one or more additional ingredients, all donating their specific functionalities to the hybrid gel. Some aspects of multifunctional nanocellulose hydrogels have also been reviewed recently.^[^ ^13^, ^142^, ^143^ ^]^

### 3.1 Background: Pure Nanocellulose Gels

As a background, we briefly discuss hydrogelation upon dispersing nanocelluloses in aqueous media without additional major components. In the molecular scale, unmodified cellulose chains have only limited water solubility because of strong inter‐ and intramolecular hydrogen bonding.^[^ ^144^ ^]^ Therein functional transformation or derivatization of the hydroxyl groups have been used to alter the water solubility.^[^ ^145^ ^]^ Polymeric cellulose derivatives such as methylcellulose, ethylcellulose, or hydroxypropyl cellulose show remarkable improvement in the water dispersibility and exhibit thermosensitive gelation.^[^ ^146^, ^147^ ^]^ Due to their biocompatibility and nontoxic nature, cellulose derivatives have been used, e.g., as emulsifiers, viscosity modifiers, wound dressing, tissue culture scaffolds, in pharmaceutical and biomedical applications, and in tissue culture studies.^[^ ^3^, ^148^ ^]^ By contrast, in the colloidal scale, nanocelluloses have a more pronounced tendency to aggregate because of hydrogen bonding and other physical interactions between them. Mechanical, chemical, or enzymatic treatments promote dispersion in aqueous media. Chemical pretreatments and functionalization often result in highly negatively or positively charged surfaces, depending on the method of modification. Functional groups such as carboxylates, sulfate half‐esters, phosphorylates and quaternary amines on nanocelluloses have been exploited to achieve colloidal stability.^[^ ^149^ ^]^ For example, as discussed in Section 2.3, TEMPO‐oxidized CNF produces nanofibrils with lateral dimensions of 3–4 nm with surface carboxylate groups, allowing suspension in aqueous media. The hygroscopic nature of cellulose, the high specific surface area and the moderately high aspect ratio of the CNF contribute to strongly interconnected networks resulting in highly viscous suspension and hydrogelation at low solid content.^[^ ^150^, ^151^, ^152^ ^]^

CNF suspensions exhibit gelation (*G*′ > *G*″, *G*′ ∝ ω^0^, and *G*″ ∝ ω^0^, where, *G′* is the storage modulus, *G″* is the loss modulus, ω is the frequency) even down to a concentration near to 0.1 wt%, i.e., the critical gelation concentration, above which the nanofibrils form interconnected networks.^[^ ^153^ ^]^ CNF hydrogels follow power law behavior, *G*′ ∝ *c^n^*, where *c* is the cellulose concentration.^[^ ^154^ ^]^ Depending on the CNF suspension, a broad range of *n* values from 2 to 5.2 have been reported in the literature.^[^ ^155^, ^156^ ^]^ Comparing to elastic and semiflexible fibrous biopolymeric gels, the *n*‐values proposed for CNF hydrogels can be considerably larger.^[^ ^157^, ^158^, ^159^, ^160^ ^]^ CNF suspensions possess shear thinning and thixotropic properties and their mechanical properties depend strongly on the concentration as well as the pH.^[^ ^5^, ^155^, ^161^, ^162^, ^163^, ^164^, ^165^ ^]^ Unlike CNFs, the rod‐like CNCs are less susceptible to gelation, unless chemically modified.^[^ ^166^ ^]^ CNCs sooner self‐assemble into chiral nematic liquid crystals above 4.5 wt%.^[^ ^168^, ^169^, ^170^ ^]^ The chiral nematic assembly can be tuned by shearing CNC encapsulated gel matrices or microfluid‐based droplets toward, e.g., soft hydrogel‐based sensors.^[^ ^171^, ^172^ ^]^ Nanocellulose hydrogels have been studied in the context of biomedical applications,^[^ ^173^, ^174^ ^]^ tissue engineering,^[^ ^175^ ^]^ cell culture,^[^ ^176^ ^]^ rheology modifier,^[^ ^177^ ^]^ and reinforcing agent.^[^ ^9^, ^10^, ^178^, ^179^ ^]^

### 3.2 Stimuli‐Responsive Nanocellulose Hybrid Gels

**Figure 5:**
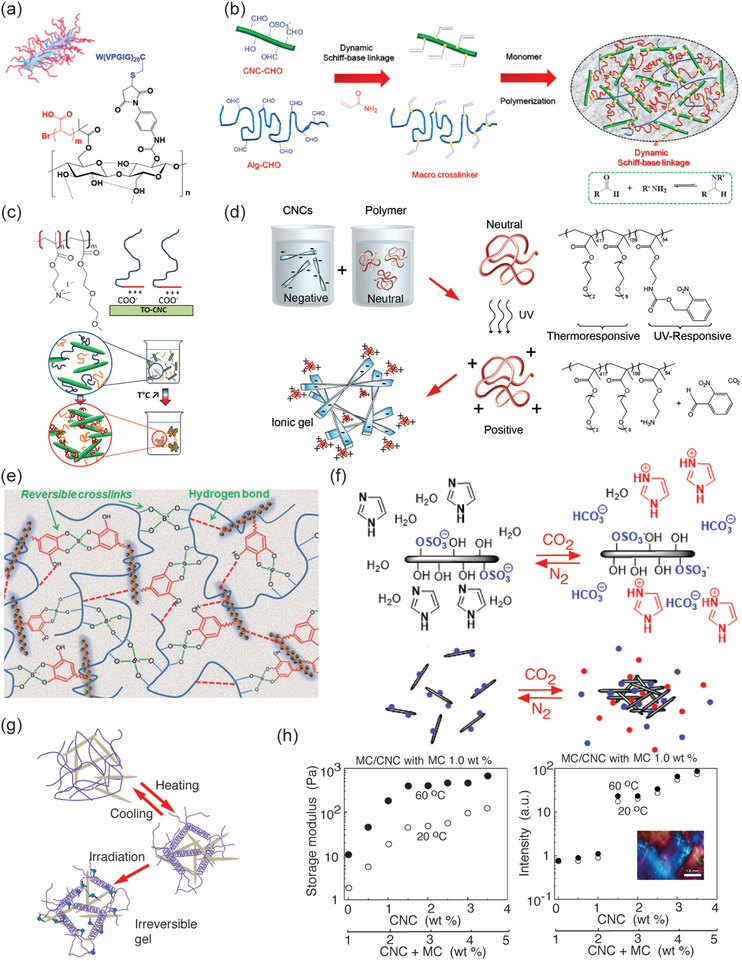
Stimulus‐responsive CNC hybrid gels and concepts thereof. a) A scheme of a CNC colloidal brush and a structure constituting of mixed PAA and elastin‐like protein brushes. Reproduced with permission.^[^ ^193^ ^]^ Copyright 2018, American Chemical Society. b) Hybrid hydrogels based on oxidized CNC and sodium alginate, reacted with amine‐containing vinyl functionalized monomers via Schiff‐base reaction, followed by in situ polymerization. Reproduced with permission.^[^ ^208^ ^]^ Copyright 2020, Elsevier. c) Electrostatic binding of PDMAEMA‐*block*‐PDEGMA onto the anionic CNC due to the cationic PDMAEMA block, leading to thermoresponsive gelation due to the PDEGMA block. Reproduced with permission.^[^ ^210^ ^]^ Copyright 2019, American Chemical Society. d) On‐demand electrostatic binding of a copolymer on CNC by UV‐triggerable cationization of part of the repeat units, where the other part allows reversible or irreversible gelation, depending on the heating times. Reproduced with permission.^[^ ^211^ ^]^ Copyright 2019, American Chemical Society. e) CNCs decorated with Ag nanoparticles and tannic acid, where mixing with PVA and borates leads to conductivity that is responsive to mechanical deformations. Reproduced with permission.^[^ ^213^ ^]^ Copyright 2019, Royal Society of Chemistry. f) CO_2_ absorption leads to responsive gelation. Reproduced with permission.^[^ ^217^ ^]^ Copyright 2018, American Chemical Society. g) CNCs decorated with methacrylate groups and mixed with gelatin methacryloyl lead to reversible gels. Upon shear orientation and interlocking by photocrosslinking renders anisotropic properties. Reproduced with permission.^[^ ^219^ ^]^ Copyright 2019, Wiley‐VCH. h) Methylcellulose with CNC allows thermally driven hybrid gels, showing percolation both in the mechanical properties and birefringence. Reproduced with permission.^[^ ^221^ ^]^ Copyright 2018, American Chemical Society (https://pubs.acs.org/doi/10.1021/acs.biomac.8b00392; further permissions related to the material excerpted should be directed to the ACS).

pH‐responsive gelation can be controlled using pure nanocelluloses by selecting their surface charges properly, such as using carboxylic acids or amination.^[^ ^180^, ^181^ ^]^ However, taken that a broader selection of stimuli‐responsive behaviors are pursued, hybrid gels turn particularly feasible, incorporating additional polymeric components that are also stimulus‐responsive to achieve multistimuli‐responsive gels. Such polymers can be covalently connected to the nanocelluloses or through nanocomposite mixtures. In the first option, the stimulus‐responsive polymers can be connected as side chains onto CNCs to form colloidal brushes (for a schematic presentation of CNC colloidal brush, see the inset of **Figure** **5**a). Poly(*N*‐isopropylacrylamide) (PNIPAm) is a classic thermoresponsive polymer, which exhibits a lower critical solution temperature (LCST) phase behavior in the aqueous media.^[^ ^182^ ^]^ Therein PNIPAm chains dissolve in water upon cooling but phase separate above 32 °C. It has been widely used in thermoresponsive applications, such as drug release, tissue cultures, and optical properties. Several chemistries are available to provide PNIPAm brushes onto nanocellulose. For example, single‐electron transfer living radical polymerization (SI‐SET‐LRP) allows to graft PNIPAm brushes onto CNCs where the effect of the grafting densities and molecular weight on the colloidal stability have been explored,^[^ ^183^, ^184^, ^185^ ^]^ The compositions also render thermoresponsive Pickering emulsions.^[^ ^186^ ^]^ SI‐ATRP,^[^ ^187^ ^]^ has extensively been used to chemically link various types of polymers brushes onto CNC (see Section 2.4), also PNIPAm.^[^ ^21^, ^76^, ^98^, ^106^, ^188^, ^189^, ^190^ ^]^ Apart from PNIPAm, tunable LCST‐behavior has been achieved by SI‐ATRP‐polymerization of poly(oligo(ethylene glycol) methacrylate) brushes onto CNC.^[^ ^191^, ^192^ ^]^ Besides synthetic polymer bushes, SI‐ATRP has been utilized to functionalize CNCs with a combination of poly(acrylic acid) (PAA) and biosynthetic elastin‐like polypeptides (ELP), where the ELP brushes render thermoresponsivity. Such materials are translucent below 20 °C and turbid above 20 °C (Figure 5a).^[^ ^193^ ^]^ Importantly, other grafting techniques are available: Poly(*N*‐isopropylacrylamide‐*co*‐acrylic acid)‐grafted cellulose nanocrystals have been prepared by reversible addition‐fragmentation chain transfer polymerizations,^[^ ^194^ ^]^ poly(di(ethylene oxide) methyl ether methacrylate) grafts using cerium ammonium nitrate initiators,^[^ ^195^ ^]^ and peptide coupling to conjugate PNIPAm‐brushes to CNCs (see Section 2.3).^[^ ^196^ ^]^

The above functionalities provide a platform for stimuli‐responsive hybrid nanocellulose gelation. CNCs with PNIPAm brushes can lead to gelation even when no covalent crosslinkers are incorporated. Such materials have been applied in functional wound healing.^[^ ^197^ ^]^ Double networks have, in general, turned relevant for increased toughness by facilitating fracture energy dissipation mechanisms.^[^ ^198^ ^]^ PNIPAm copolymerization with PAA and mixtures with poly(vinyl alcohol) (PVA) and CNC afford double‐network hydrogels upon photo‐crosslinking.^[^ ^199^ ^]^ Aqueous mixtures of CNCs and PNIPAm form hydrogels which also exhibit tunable optical properties.^[^ ^200^ ^]^ Therein, above the LCST, the hydrogel films show promoted light scattering, thus expressing “whiteness,” whereas, below the LCST, the hydrogels show translucency. This concept can be extended to achieve even stronger switchable whiteness using other polysaccharide component (instead of CNCs) such as agarose in PNIPAm hybrid gels, upon finally removing the agarose component.^[^ ^201^ ^]^

In another approach for thermoresponsive behavior, film‐like bilayer actuators are well known in science and technology, usually based on asymmetric thermal expansion and bending. Bilayer‐like actuation is observed even in paper sheets upon anisotropic water immersion from one side, which allows transient bending.^[^ ^202^ ^]^ Such a phenomenon is widely observed in biological systems, such as pine cones.^[^ ^203^, ^204^ ^]^ The bilayer actuation concept has been demonstrated recently in wooden constructs.^[^ ^205^ ^]^ Related to stimuli‐responsive CNC hydrogels, bilayer hydrogel actuators have been obtained based on two hybrid hydrogel layers, viz. CNC/PNIPAm) and CNC/poly(*N*‐hydroxyethyl acrylamide), which form a bilayer structure.^[^ ^206^ ^]^ Similar to PNIPAm, thermoresponsive behavior is also observed in aqueous poly(*N*‐vinyl caprolactam), leading to reversible aggregation upon heating.^[^ ^207^ ^]^ Covalently crosslinked hybrid hydrogel gels have been prepared using CNC as reinforcing agent in sodium alginate. Therein, their partial oxidization renders aldehyde groups allowing Schiff‐base reactions followed by acrylamide polymerization, where the CNC acts as a reinforcement (Figure 5b).^[^ ^208^ ^]^ Thermoresponsive behavior and drug‐release are achieved when incorporating di(ethylene glycol) methyl ether methacrylate and (ethylene glycol) methyl ether methacrylate monomers in the polymerizations. In another approach, hybrid supramolecular hydrogels consisting of hydroxypropyl methylcellulose and CNCs with short side chain brushes which were hydrophobically modified by octyl side chain is reported. Upon heating from room temperature up to 80 °C, the storage modulus of the hybrid gel increases by an order of magnitude.^[^ ^209^ ^]^

Beyond covalent polymer brushes on CNCs, also physical bindings are used, allowing modularity. Therein diblock copolymers are feasible, involving a block that binds onto CNC and the other end block being responsive. For example, TEMPO‐oxidized CNC was complexed electrostatically with a diblock copolymer consisting of a poly(2‐(dimethylamino)ethyl methacrylate) (PDMAEMA) cationic polyelectrolyte block and a poly(di(ethylene glycol) methyl ethermethacrylate) (PDEGMA) thermoresponsive block. Thermoresponsive gelation is achieved taken an excessive amount of the diblock copolymer (Figure 5c).^[^ ^210^ ^]^ Even dual‐stimuli‐responsive copolymers have been used to allow dual stimuli‐responsive CNC hybrid gels (Figure 5d).^[^ ^211^ ^]^ Therein pristine poly(2‐((2‐nitrobenzyl)oxycarbonyl)aminoethyl methacrylate)‐*rnd*‐[poly(di(ethylene glycol) methyl ether methacrylate)‐*rnd*‐poly(oligo(ethylene glycol) methyl ether methacrylate)] is noncharged and allows homogeneous mixing with the negatively charged CNCs. Upon ultraviolet radiation, nitrobenzyl groups are cleaved from the poly(2‐((2‐nitrobenzyl)oxycarbonyl)aminoethyl methacrylate) part, thus making it cationic and allowing stimulus‐switchable on‐demand binding onto CNC. On the other hand, the poly(di(ethylene glycol) methyl ether methacrylate)‐*rnd*‐poly(oligo(ethylene glycol) methyl ether methacrylate) part therein leads to LCST thermoreversible gelation upon heating to 60 °C. Short heating times allow thermoreversible gelation, whereas long heating leads to irreversible gelation.

Tannic acid is proven to be a useful compound in general to mediate interactions and adhesion.^[^ ^212^ ^]^ Inspired by thereof, hybrids of CNCs decorated with tannic acid and silver nanoparticles, when combined with PVA via borate ester crosslinks, lead to responsive and self‐healing hybrid hydrogels (Figure 5e). In combination with the high stretchability in excess of 4000%, it allows skin‐mimetic materials for devices.^[^ ^213^ ^]^ CNCs were also used to reinforce composite hydrogels containing peptide–peptoid copolymers and polysaccharides based on acylhydrazone linkages. Peptide–peptoid copolymers were prepared using sarcosine and l‐glutamic acid γ‐benzyl ester, the benzyl units were substituted with hydrazide groups. Upon mixing with CNC and aldehyde‐modified sodium alginate, pH‐responsive gelation was achieved, intended for drug release or tissue scaffolding.^[^ ^214^ ^]^ Tannic acid has been used for compositions with CNCs, PAA chains, and Al^3+^‐ions in order to construct covalent polymer networks. Reversible dynamic multiple coordination interactions allow self‐healing and adhesiveness as well as strain sensitivity for flexible strain sensors.^[^ ^215^ ^]^ Finally, as reviewed already early,^[^ ^216^ ^]^ carbon dioxide reacts reversibly with different amines, which has led to a wide variety of approaches for hydrogelation and organogelation based on the CO_2_‐stimuli. In relation to CNCs, CO_2_‐switchable hydrogels have been obtained by adding imidazole to an aqueous suspension (Figure 5f).^[^ ^217^ ^]^

Classically CNCs form cholesteric liquid crystals.^[^ ^15^, ^167^, ^168^, ^169^, ^170^, ^218^ ^]^ Upon strong shearing and interlocking of the structure by photopolymerization of a hybrid gel matrix phase, monodomain nematic alignment can be achieved and stabilized.^[^ ^171^ ^]^ Such anisotropic networks can be reversibly swollen. Hydrogels with anisotropic swelling have been achieved using cellulose nanocrystal methacrylate and gelatin methacryloyl exploiting microfluidic extrusion, followed by cooling down and photocrosslinking (Figure 5g).^[^ ^219^ ^]^

Inspired by the plant cell wall scaffolds, where the cellulose microfibrils are held together by hemicellulose chains (see Section 2.1), hybrid hydrogels based on CNC and methylcellulose (MC) were constructed.^[^ ^220^, ^221^ ^]^ At room temperature, MC exists as random coils whereas above LCST, it aggregates into persistent fibrils with a lateral dimension of ≈14 nm^[^ ^222^ ^]^ and length of several hundreds of nanometers. In MC–CNC hybrids, gelation was observed upon heating and when the temperature of 60 °C was approached, strain hardening was observed under oscillatory rheological measurements. The modulus is tunable by adjusting the composition. Interestingly, the hybrid gels showed birefringence even at low CNC concentrations in the range <1.5 wt%, i.e., considerably below the critical liquid crystal concertation of pure CNCs (Figure 5h). The mechanical properties of the hybrid gels increase until 1.5% of CNC, above which there was a plateau, thus suggesting a percolation‐like behavior both in birefringence and storage modulus.^[^ ^221^ ^]^

Finally, actuation can also be achieved by electric fields, based on CNCs photopolymerized with Na‐4‐vinylbenzenesulfonate, 2‐hydroxyethylmethacrylate, and acrylonitrile. Interestingly, actuation is achieved using very low voltages down to 5 mV cm^−1^, i.e., below the water electrolysis limit, thus expanding the practical usefulness.^[^ ^223^ ^]^

**Figure 6:**
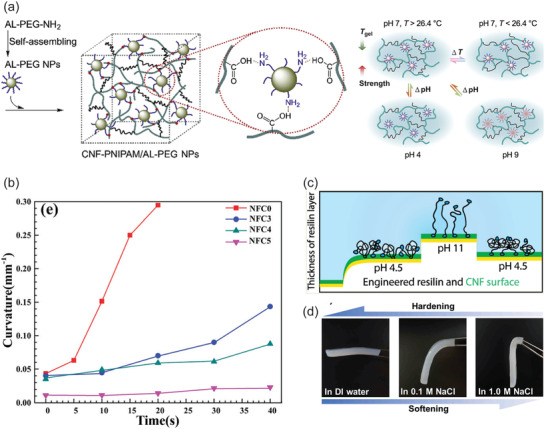
Stimulus‐responsive CNF hybrid gels. a) TEMPO‐oxidized CNFs and PNIPAm incorporating alkaline lignin nanoparticles decorated with poly(ethylene glycol) ligands with terminal amines lead to thermally and pH‐driven responsive hybrid gels. Reproduced with permission.^[^ ^227^ ^]^ Copyright 2019, Elsevier. b) Bilayer actuators with CNF facilitate bending due to thermally controlled anisotropic swellings. Reproduced with permission.^[^ ^229^ ^]^ Copyright 2018, Royal Society of Chemistry. c) Hybrid gels based on CNFs and genetically engineered adhesive resilin fusion protein lead to pH‐dependent responsivity. Reproduced with permission.^[^ ^231^ ^]^ Copyright 2017, American Chemical Society (https://pubs.acs.org/doi/10.1021/acs.biomac.7b00294; further permissions related to the material excerpted should be directed to the ACS). d) Ionic strength can be used as a stimulus for responses of CNF containing hybrid hydrogels. Reproduced with permission.^[^ ^232^ ^]^ Copyright 2018, Royal Society of Chemistry.

Moreover, CNF hybrid gels possess stimuli‐responsive behaviors and several of them display multifunctional properties. CNFs can be modified with polymer brushes, in analogy with CNCs. However, as CNFs are not fully crystalline, subtleties can arise: SI‐ATRP provides chemistries to chemically connect brushes on CNF, though certain degree of degradation processes are observed, presumably from the CNF disordered sites.^[^ ^107^ ^]^ Three‐component Passerini reactions have been used to chemically link polymer brushes on CNF, such as PNIPAm chains.^[^ ^224^ ^]^ Freeze‐casting has been used to prepare aligned aqueous mixtures of CNF and *N*‐isopropylacrylamide monomers, followed by photopolymerization, leading to thermoresponsivity with a large swelling ratio.^[^ ^225^ ^]^ The composition was considered toward the controlled release of antibacterial components into biofilms. TEMPO‐oxidized bamboo CNFs hybrids with PNIPAm brushes have been synthesized with free radical polymerization toward thermoresponsive swelling with LCST behavior.^[^ ^226^ ^]^ The gels consisting of TEMPO‐oxidized CNFs and PNIPAm can incorporate additional alkaline lignin nanoparticles decorated with poly(ethylene glycol) ligands with terminal amines. The storage modulus increases upon increased temperature with its maximum storage modulus value at pH = 6.5, due to the control of the amine/carboxylic acid supramolecular interactions (**Figure** **6**a), leading to both thermoresponsivity and pH‐responsivity.^[^ ^227^ ^]^

Bilayer actuators can also be prepared using CNF similar to that of wood and CNC based actuators. In the simplest case, micrometer thick CNF films can undergo reversible binding upon exposing humidity from one side of the film, thus leading to anisotropic humidity‐driven swelling.^[^ ^228^ ^]^ CNF‐based bilayer multicomponent hybrid hydrogels were constructed, where one layer consists of graphene oxide, hectorite clay, CNF, PVA, and PNIPAm and the other layer was similar but excluding graphene oxide and CNF. The resulting hybrid bilayer hydrogels display thermoresponsive bending and actuation (Figure 6b).^[^ ^229^ ^]^

Hybrid hydrogels using cationic star‐block copolymers of poly(di(ethylene glycol)methyl ether methacrylate) (PDEGMA) and quaternized poly(2‐(dimethylamino)ethyl methacrylate) and TEMPO–CNF were studied for their thermoresponsive behavior.^[^ ^230^ ^]^ Hybrid gels can be prepared using CNFs and genetically engineered resilin fusion protein decorated at both ends, by cellulose binding modules, allowing interaction with the CNFs. The conformation behavior of the protein component was found to be pH‐responsive (Figure 6c).^[^ ^231^ ^]^ Finally, ionic strength can be used as a stimulus to allow responses. Hydrogels are shown to soften reversibly with the increase in ionic strength based on bacterial cellulose and sodium polystyrene sulphonate (Figure 6d).^[^ ^232^ ^]^

### 3.3 Shape‐Memory Nanocellulose Hybrid Hydrogels

**Figure 7:**
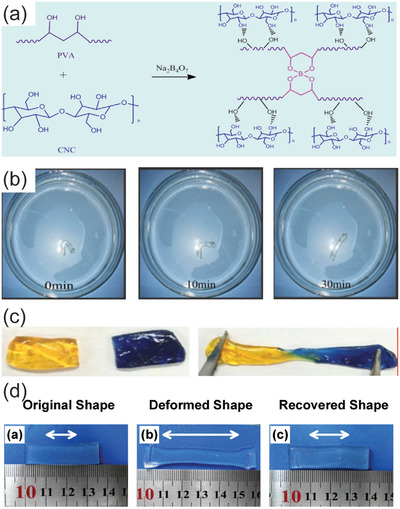
Shape‐memory effects related to nanocelluloses. a) Chemical structures of PVA and CNC and borate crosslinked hybrid system. b,c) Shape‐memory (b) and self‐healing (c) for such materials. a–c) Reproduced with permission.^[^ ^234^ ^]^ Copyright 2020, Elsevier. d) Shape‐memory states of TEMPO‐oxidized CNF, polyacrylamide, and gelatin. Reproduced with permission.^[^ ^235^ ^]^ Copyright 2017, Elsevier.

In addition to simple responsive behavior, more complicated responses are accessible for hybrid nanocellulose hydrogel systems, such as shape‐memory effects. In shape‐memory materials, the system is first kinetically locked to a temporary state by the first stimulus and then released from the kinetic trap by a second stimulus to regain the equilibrium shape.^[^ ^233^ ^]^ Shape‐memory materials have been constructed based on CNC with PVA hybrid hydrogels using reversible borate‐ester bonds (**Figure** **7**a,b).^[^ ^234^ ^]^ The composition also allows self‐healing (Figure 7c). TEMPO‐oxidized CNF, polyacrylamide, and gelatin were used to construct shape‐memory hybrid gels (Figure 7d). The shape memory effect, was demonstrated by preparing a rod‐like hydrogel specimen. The rod‐like structure was then bent to a new V‐shaped object and stabilized by physical crosslinking at 55 °C for 30 min. Upon reimmersing the V‐shaped structure in water, the original shape was regained.

### 3.4 Self‐Healing of Nanocellulose Hybrid Hydrogels

**Figure 8:**
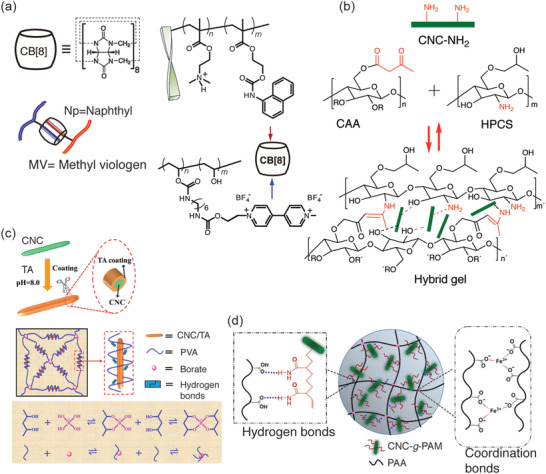
Self‐healing CNC‐containing hybrid gels. a) Cucurbituril, CB[8] can selectively host both methyl viologen and naphthyl groups, thus allowing rapidly exchangeable supramolecular crosslinks to mediate self‐healing. Reproduced with permission.^[^ ^236^ ^]^ Copyright 2014, Wiley‐VCH. b) Self‐healing hybrid hydrogels of amino‐modified CNCs, cellulose acetoacetate, and hydroxypropyl chitosan. Reproduced with permission.^[^ ^239^ ^]^ Copyright 2018, Springer Nature. c) CNFs coated with tannic acid, mixed with PVA, and crosslinked with borates for self‐healing hybrid hydrogels. Reproduced with permission.^[^ ^240^ ^]^ Copyright 2019, American Chemical Society. d) Fe^3+^ coordination mediated hybrid hydrogels based on polyacrylamide functionalized CNC and PAA. Reproduced with permission.^[^ ^241^ ^]^ Copyright 2019, Springer Nature.

An early demonstration of a CNC‐based self‐healing hybrid gel system was reported using dynamic and selective three‐component supramolecular host–guest interactions.^[^ ^236^ ^]^ The nanocomposite hydrogel involves two guests viz.: i) PVA functionalized with methyl viologens (MVs) methacrylate (PVA‐MV), and ii) CNC grafted with poly(diethylaminoethyl methacrylate) (DMAEMA), and naphthyl functionalized methacrylate (NpMA), i.e., CNC‐*g*‐P(DMAEMA‐*rnd*‐NpMA). The third component, cucurbit‐[8]‐uril (CB[8]) functions as a supramolecular host (**Figure** **8**a). Because of its large cavity (8.8 Å), selective guest encapsulation, and high equilibrium binding constants (*K*_eq_ ≈ 10^12^ m^−1^), mixing equimolar concentration (Np:MV 1:1) of the guests with CB[8] showed gels with a high storage modulus of 14.3 kPa and self‐healing even after uncommonly prolonged storage of the material for four months without seeming passivation. The concept was extended to prepare a four‐component hybrid hydrogel comprising heteroternary supramolecular system and colloidal CNF as reinforcing moiety.^[^ ^237^ ^]^ The supramolecular hybrid hydrogels consisted of naphthyl appended hydroxyethyl cellulose (HEC‐Np) and viologen functionalized styrene derivative as guests in the presence of CB[8]. It indicates self‐healing in rheological experiments. In general, interactions that allow reversible bond formation can promote self‐healing. Furyl‐decorated CNCs and dimaleimide poly(ethylene glycol) lead to self‐healing hydrogels via thermally reversible covalent Diels–Alder reactions to form exchangeable crosslinks.^[^ ^238^ ^]^ Dynamic enamine bonds facilitate self‐healing under acidic conditions. A hydrogel was achieved based on amino‐modified CNCs, cellulose acetoacetate, and hydroxypropyl chitosan under physiological conditions (Figure 8b).^[^ ^239^ ^]^ Tannic acid is a useful component also in CNF‐based self‐healing materials. CNFs coated with tannic acid lead to self‐healing upon mixing with PVA crosslinked with borates (Figure 8c).^[^ ^240^ ^]^ Metal coordination, for example, Al^3+^‐ion mediated coordination bonds afford hybrid hydrogels with self‐healing property and adhesion properties.^[^ ^215^ ^]^ Tannic acid when used with Ag nanoparticles and a mixture of PVA with borate ester crosslinks leads to highly stretchable self‐healing CNC‐containing hybrid hydrogels.^[^ ^213^ ^]^ Acrylic acid was polymerized in mixtures of CNCs grafted using polyacrylamide brushes (CNC‐*g*‐PAm). Fe^3+^‐ions and hydrogen bonds mediate supramolecular interactions; viz., i) between PAA chains and polyacrylamide grafted on CNCs and ii) between PAA chains (Figure 8d). Due to two types of interactions, tough and self‐healing hybrid gels were achieved.^[^ ^241^ ^]^ Hybrid hydrogels were constructed based cellulose nanocrystals grafted by poly(glycerolmonomethacrylate) in the mixtures of poly(*N,N*‐dimethylacrylamide)‐*rnd*‐poly(3‐acrylamidophenylboronic acid) statistical copolymers. Self‐healing was achieved based on the reversible boronic ester interactions.^[^ ^242^ ^]^ Even more complex supramolecular binding motifs can be used, such as ureidopyrimidinone (UPy) which undergoes four hydrogen bonds.^[^ ^243^ ^]^ CNC was modified by an UPy and mixed with PVA to form hybrid self‐healing hydrogels.^[^ ^244^ ^]^

Similar to CNC‐based materials, CNF allows self‐healing hybrid hydrogels. Incorporation of PVA and borax‐mediated exchangeable metal coordinations provides a versatile approach in connection of CNF.^[^ ^245^ ^]^ Lignin nanoparticles could be additionally incorporated in the compositions to improve the moduli values.^[^ ^246^ ^]^ One of the useful properties of PVA is that freeze‐thawing generates local PVA crystalline domains. The crystalline domains acts as physical crosslinks, thus improving the mechanical properties of the self‐healing CNF‐containing hybrid hydrogels.^[^ ^247^ ^]^ In another approach, 3,3‐dithiobis(propionohydrazide) facilitates bonds with dialdehyde carboxymethyl cellulose to render self‐healing CNF hybrid gels.^[^ ^248^ ^]^

**Figure 9:**
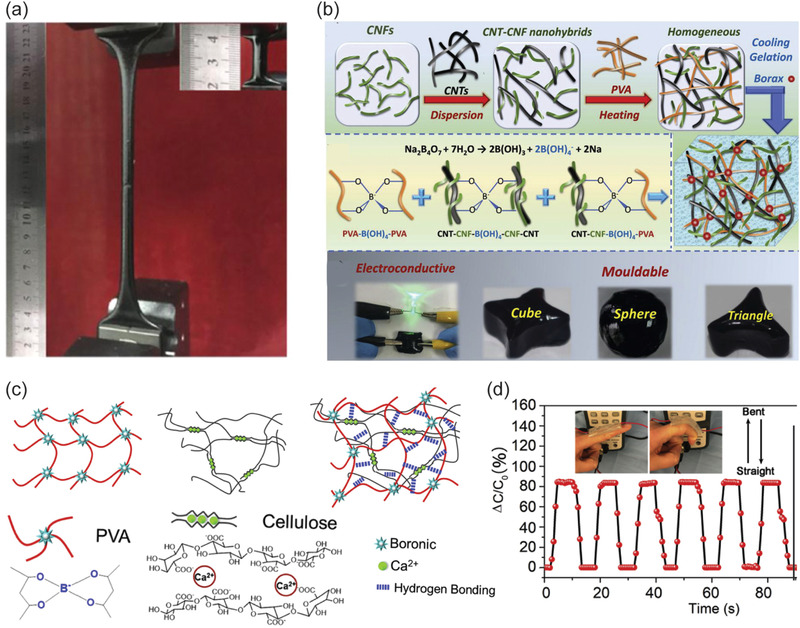
Self‐healing hydrogels based on CNF containing hybrid hydrogels. a) CNF as a dispersing medium for multiwalled carbon nanotubes in combination with hydrophobically associated polyacrylamide for self‐healing hybrid gels. Reproduced with permission.^[^ ^250^ ^]^ Copyright 2018, American Chemical Society (https://pubs.acs.org/doi/10.1021/acsami.7b18700; further permissions related to the material excerpted should be directed to the ACS). b) Carbon nanotube dispersed in water by TEMPO‐oxidized CNF, polyaniline polymerized on the CNF/carbon nanotube scaffolds, and mixing with PVA with borax crosslinking, leading to multifunctional behavior. Reproduced with permission.^[^ ^251^ ^]^ Copyright 2019, Elsevier. c,d) Hybrid gels composed of CNFs with PVA crosslinked with Ca^2+^ and borax (c) and their time response (d). c,d) Reproduced with permission.^[^ ^257^ ^]^ Copyright 2019, Elsevier.

A particularly relevant additional functionality related to self‐healing concerns electrically conductive hydrogels. In such systems, the mechanical healing is expected to be followed by the restoration of the electrical conductivity upon healing. Several approaches have been reported by incorporation electrically conducting conjugated polymers or nanocarbons. Accordingly, CNFs have been decorated with conjugated electrically conducting polymer polypyrrole where the mixtures combine also PVA and borax, thus leading to self‐healing conductive hydrogels.^[^ ^249^ ^]^ CNFs can be used as a dispersing medium for multiwalled carbon nanotubes (MWCNTs) in aqueous media. Combination of CNFs, MWCNTs, and polyacrylamide leads to self‐healing hybrid gels rendering electric conduction and even electromagnetic shielding (**Figure** **9**a).^[^ ^250^ ^]^ Upon increasing the complexity, electroconductive hybrid hydrogels were shown based on TEMPO‐oxidized CNF, which promotes carbon nanotube dispersion in water, optionally followed by conjugated polymer polyaniline, which is in situ polymerized on the CNF/carbon nanotube scaffolds. In addition, the composition involves PVA upon mixing chains with borax crosslinking. The systems show self‐healing, high stretchability, and the compositions reach relatively high electronic conductivity 0.15 S cm^−1^, allowing flexible electronic wiring (Figure 9b).^[^ ^251^, ^252^, ^253^ ^]^ A related approach involves the dispersion of graphene using CNF, additionally incorporating PVA‐borax physical network to form the hydrogel.^[^ ^254^ ^]^ Besides, Fe^3+^ ions can be used for crosslinking, as shown in mixtures of TEMPO oxidized CNFs and PAA, leading to promoted mechanical strength, toughness, and self‐healing properties.^[^ ^255^ ^]^ Polypyrrole can be used for conduction, where TEMPO‐oxidized CNF mixtures with PAA with the presence of ferric ions as ionic crosslinkers lead to conducting hybrid self‐healing gels.^[^ ^256^ ^]^ Borax and Ca^2+^ crosslinked hybrid gels were prepared using polypyrrole‐coated CNCs and CNFs as reinforcements with PVA showing self‐healing hydrogels, and artificial skin‐like sensors (Figure 9c).^[^ ^257^ ^]^ Multifunctional properties were achieved, allowing self‐healing, conductivity, sensing, and also adhesion.^[^ ^258^ ^]^ Even magnetic properties can be incorporated to hybrid gels. Agarose hydrogels containing CNF reinforcements decorated with conjugated polymer polypyrrole/Fe_3_O_4_ facilitate conductivity and magnetically responsive materials. The hydrogels are healable upon thermal treatment.^[^ ^259^ ^]^ In another approach, self‐healing magnetic hybrid gels are achieved by CNF coated with polyaniline mixed with PVA and MnFe_2_O_4_ nanoparticles for hybrid gels. They were multifunctional showing self‐healing, magnetic and conductive property.^[^ ^260^ ^]^

Hybrid gels can also render promoted heavy metal ion absorption capacity. Hydrogels of TEMPO‐oxidized CNF and polyacrylamide self‐heal and show high Cu‐ion absorption toward robust heavy metals absorbing filters.^[^ ^261^ ^]^ Finally, self‐healing chiral photonic films fabricated by the coassembly of CNCs were prepared with a boronic ester crosslinked PVA–PAm hydrogel.^[^ ^262^ ^]^

### 3.5 Nanocellulose‐Based Hybrid Gels with Gluing and Adhesive Properties

Adhesion and gluing exploiting the properties of nanocelluloses has been reviewed previously in the literature.^[^ ^263^ ^]^ However, it has aroused increasing experimental interest more recently. Here we address selected approaches, mainly focusing on hybrid gelation. In a classic approach, nanocelluloses have been used to reinforce traditional glues, such as wood adhesives, as reviewed recently.^[^ ^264^ ^]^ 3‐Aminopropyltriethoxysilane‐modified CNC was explored to modify urea‐formaldehyde adhesive for the production of medium‐density fiberboard.^[^ ^265^ ^]^ Moreover, CNF has been used as a reinforcing additive in urea‐formaldehyde resin formulations.^[^ ^266^ ^]^ Epoxy resins have been modified using CNCs and in combination with calcium sulfate fillers, showing improvement of the adhesive properties,^[^ ^267^ ^]^ as well as allowing pressure sensitive acrylic glues.^[^ ^268^ ^]^ CNC‐based latex particles for adhesion and gluing were achieved through adsorption with MC to produce MC‐coated CNCs.^[^ ^269^ ^]^ On the other hand, CNFs were dispersed into commercial polyvinyl acetate and starch adhesives, and explored in wood joining using single‐lap joints. Even a small addition of CNF induced considerable improvements in adhesive properties.^[^ ^270^ ^]^ In cotton‐seed protein‐based adhesives, ≈2% CNF gave a 22% improvement in dry adhesive strength.^[^ ^271^ ^]^

**Figure 10:**
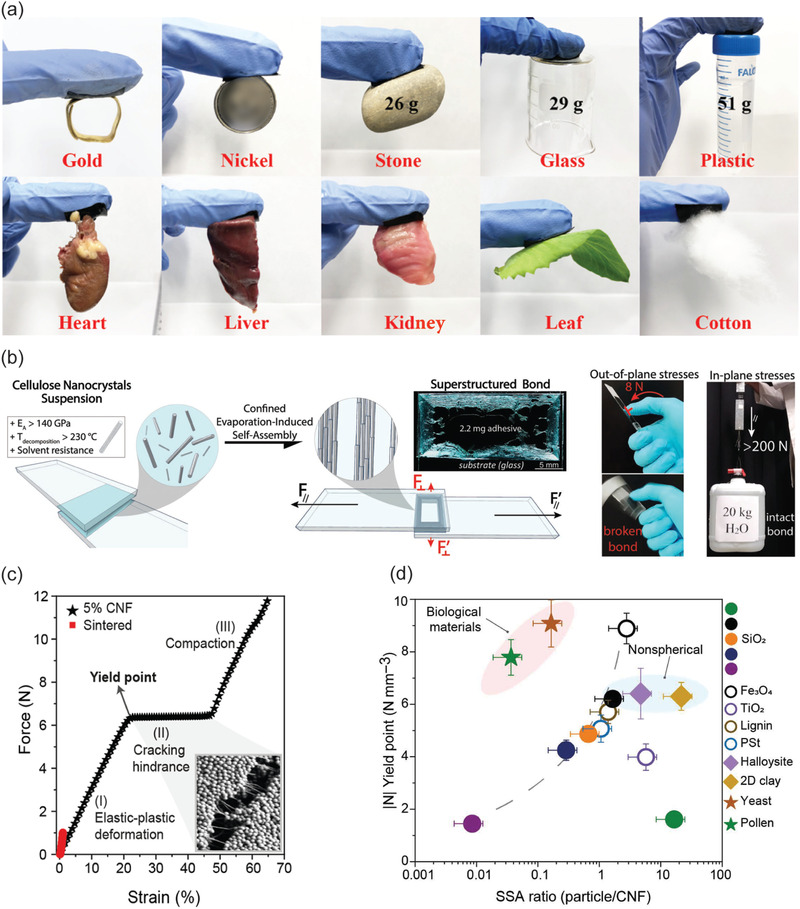
Gluing based on nanocellulose. a) Hybrid gels by conjugated polypyrrole‐coated nanocellulose with PVA and Fe^3+^‐mediated coordinative crosslinks provide adhesion to many substances. Reproduced with permission.^[^ ^258^ ^]^ Copyright 2019, American Chemical Society. b) Evaporation‐induced deposition of CNC for anisotropic gluing layers leads to strong adhesion in‐plane but weak adhesion out‐of‐plane. Reproduced with permission.^[^ ^273^ ^]^ Copyright 2020, Wiley‐VCH. c) CNF allows high cohesion between particles upon their gluing. d) Scaling of the paraparticle (SPs) robustness as a function of their respective specific surface area (SSA) ratio. c,d) Reproduced with permission.^[^ ^274^ ^]^ Copyright 2020 The Authors, published by AAAS. Reprinted/adapted from ref. ^[^ ^274^ ^]^ © The Authors, some rights reserved; exclusive license American Association for the Advancement of Science. Distributed under a Creative Commons Attribution Non‐Commercial License 4.0 ( CC BY‐NC http://creativecommons.org/licenses/by‐nc/4.0/).

There are recent efforts to combine multiple functions in gluing based on nanocelluloses. Tannic‐acid‐coated CNCs are combined with PVA involving borax dynamic networks to achieve a combination of high toughness, self‐healing, adhesion and strain‐stiffening properties.^[^ ^240^ ^]^ Hybrid gels were prepared using conjugated polypyrrole‐coated CNCs and CNFs, in order to reinforce PVA with Fe^3+^ mediated coordinative crosslinks to provide adhesion (**Figure** **10**a).^[^ ^258^ ^]^ Similarly, Al^3+^‐ion mediated coordination bonds allow hybrid hydrogels with adhesion.^[^ ^215^ ^]^ CNF hybrid hydrogels with PNIPAm facilitate reversible optical, bioadhesion, and thermal performance, making them suitable to be used as durable temperature‐sensitive sensors and functional biomedical devices.^[^ ^272^ ^]^ Recently gluing was achieved using evaporation‐induced self‐assembly of CNCs. Through this process, CNCs become aligned, thus providing anisotropic adhesion, offering strong gluing of a wealth of materials (Figure 10b).^[^ ^273^ ^]^ Finally, CNFs also afford gluing, as manifested upon gluing several types of particles within a strong network (Figure 10c,d).^[^ ^274^ ^]^ The above literature examples were discussed mainly in the context of gel‐like glues. However, situation changes when gluing of gels are involved. For such materials the adhesion is strongly dependent on whether hard or soft materials are glued. Related to soft and biomaterial gluing, an approach has been presented where colloidal particles capable of exchangeable fracture energy dissipating physical interactions with material to be glued provide secure gluing. While the approach was mainly studied in relation to silica nanoparticles, the approach also works when CNCs were used instead of silica nanoparticles.^[^ ^275^ ^]^

### 3.6 Hybrid Nanocellulose Gels in Medical Applications

Before focusing on specific medical applications more closely, we first discuss in general some recent miscellaneous medical uses of nanocellulose gels. The research in this field has been reviewed recently.^[^ ^176^, ^276^ ^]^ Hybrid hydrogels based on bacterial cellulose nanofibers (BCNFs) and sodium alginate show pH and electric field induced swelling offering stimuli‐responsive drug release.^[^ ^277^ ^]^ CNCs and hyaluronic acid form injectable hybrid hydrogels in situ, in which CNCs enhance the stability of the materials against hydrolytic and enzymatic degradation. The concept is promising for the controlled release of growth factors.^[^ ^278^ ^]^ TEMPO‐oxidized CNF and PNIPAm hybrid hydrogels facilitate temperature‐controlled drug release because of the thermosensitive nature.^[^ ^279^ ^]^ Two oppositely charged CNFs have been explored for pH‐dependent release of doxorubicin.^[^ ^280^ ^]^ TEMPO‐oxidized CNFs and cationic guar gum form hybrid gels stabilized by electrostatic interactions. The hybrid gels show self‐healing and they were studied for drug release in a simulated gastrointestinal tract conditions using bovine serum albumin (BSA) as a model protein drug.^[^ ^281^ ^]^ CNFs decorated with tannic acid mixed with PVA and borax crosslinking allow self‐healing, antioxidant, and antimicrobial properties.^[^ ^282^ ^]^ CNC–chitosan hybrid hydrogels have been studied for controlled enzymatic degradation and release of BSA in simulated intestinal tract conditions.^[^ ^283^ ^]^ Photoresponsive hybrid gels were prepared using a mixture alginate, TEMPO‐oxidized CNFs, and polyacrylamide having Fe^3+^ ion crosslinks and *N,N′‐*methylenebis‐acrylamide covalent crosslinkers, and tested for drug release.^[^ ^284^ ^]^ The application of nanocellulose hybrid gels in wound healing has been extensively pursued and reviewed previously.^[^ ^285^ ^]^ The effect of CNF chemical treatments have been explored for pH‐sensitive hydrogel design.^[^ ^286^ ^]^ Different types of hemicelluloses, i.e., galactoglucomannan, xyloglucan, and xylan were introduced into the hybrid gels of CNFs. The compositions containing xyloglucans turned particularly effective.^[^ ^287^ ^]^ Hybrid hydrogels were constructed based on carboxylated CNF by introducing aminated silver nanoparticles and gelatin, showing high biocompatibility and wound healing efficacy.^[^ ^288^ ^]^ TEMPO‐oxidized CNFs were used to construct hybrid gels with polydopamine with Ca^2+^ ions as crosslinkers. pH and near‐infrared (NIR)‐responsive hydrogels were achieved with controlled drug releasing property, antibacterial and mechanical properties.^[^ ^289^ ^]^ Hybrid gels consisting of bacterial cellulose and PAA allow wound dressing, drug release, and pH‐dependent responses.^[^ ^290^ ^]^ Dialdehyde bacterial cellulose hybrid with chitosan allows hydrogels with self‐healing, antibacterial and wound healing properties.^[^ ^291^ ^]^

### 3.7 Nanocellulose Hydrogels in Tissue Engineering Scaffolds

Historically, physiologically irrelevant and stiff materials such as polystyrene and glass as flat 2D culture setup up have been used for cell culture studies. Such 2D platforms are simple, and, however, at the same time lead to numerous artifacts due to flattened cells, limited cell–cell communication, cell polarization, uncontrolled change in phenotypes as well as nonoptimal response to drug candidates.^[^ ^292^, ^293^, ^294^ ^]^ Therefore, cell and tissue culture systems that mimic the physiological 3D environment with appropriate chemical, mechanical and biological queues, either alone or in combination are needed to bridge the gap between conventional culturing methods and the complexity in native cell environment. Hydrogels have been studied for a range of cell culture applications as a 3D scaffold or extracellular matrix (ECM) mimics.^[^ ^295^ ^]^ 3D hydrogel scaffolds have revealed some of the essential biochemical and mechanical aspects that regulate cell behavior, differentiation and proliferation that are not possible using traditional 2D culture platforms.^[^ ^296^ ^]^ While selecting a hydrogel matrix, the factors such as gel mechanical properties (elasticity), chemical composition, adhesive/binding sites, porosity and stability under culture conditions are considered. Therefore, biomaterials such as collagen, fibrin and other basement membrane protein‐based matrices have been successfully utilized. However, these animal‐based ECM materials have several challenges due to batch to batch variation, the presence of unknown growth factors and inability to tune their mechanical properties.^[^ ^296^, ^297^, ^298^, ^299^ ^]^ Therefore, recent efforts have been made to achieve ECM mimics using non‐animal‐based materials. Wherein, other plant and microbial‐based biopolymers, their derivatives and synthetic polymers have been used with or without chemical modification.

CNF hydrogels, due to their small fiber diameter, large surface to volume ratio, and possibilities for surface functionalization, are favorable for cell culture. The controllable porosity of CNF hydrogels also useful for efficient cellular infiltration and nutrient diffusion, therefore, utilized in cell culture, stem cell maintenance, and 3D differentiation of progenitors.^[^ ^300^ ^]^ Nanocellulose hydrogels are biocompatible and physiologically inert, but may pose challenges to in‐depth tune the mechanical properties in comparison to the best synthetic polymer gels. Moreover, CNFs are not continuous like fiber scaffolds prepared using electrospinning. Therefore, CNF 3D scaffolds via freeze‐drying or supercritical drying, protonation, crosslinking using metal ions have been used. Furthermore, nanocellulose lacks the adhesive sites necessary for cell signaling and migration. Hence, further modification is required to utilize them in culture platforms. Dynamic Schiff base formation has been utilized to prepare CNF‐chitosan injectable self‐healing hydrogels for neural regeneration.^[^ ^301^ ^]^ Hybrid CNC–PVA hydrogels prepared using freeze‐thawing offer tunable mechanical properties and are suitable for tissue culture applications.^[^ ^302^ ^]^ CNF‐based thixotropic gels are explored for encapsulation of human breast cancer cells and mouse embryonic stem cells.^[^ ^181^ ^]^ Hybrid gels of CNC with poly(caprolactone)‐*block*‐poly(ethylene glycol)‐*block*‐poly(caprolactone) were used in cell culturing applications.^[^ ^303^ ^]^ Next we will focus on selected recent examples on nanocellulose based scaffolds.

**Figure 11:**
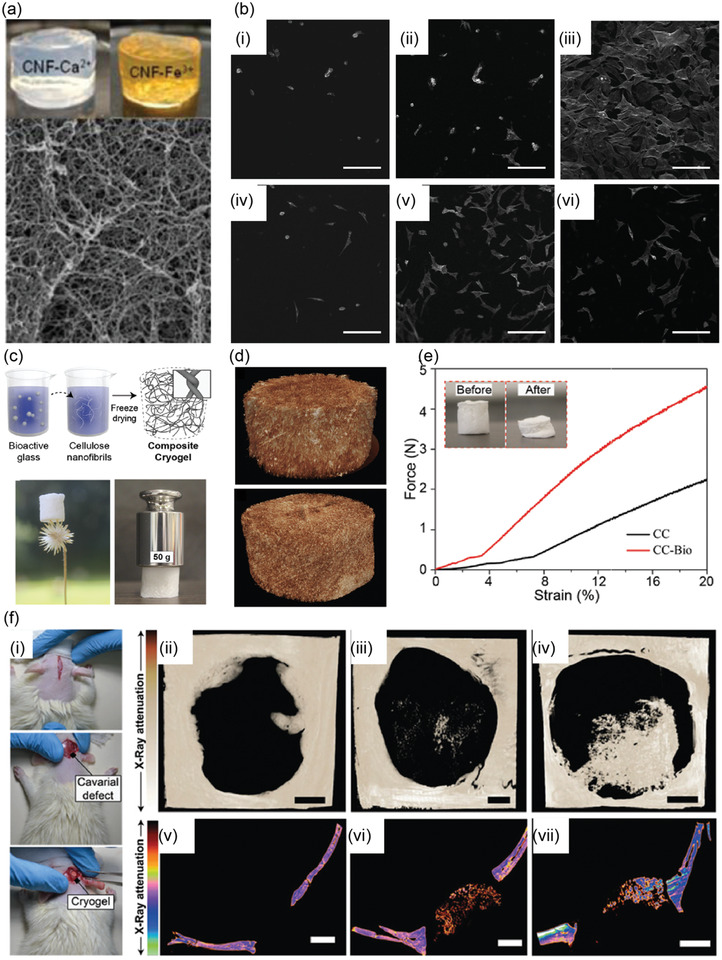
Nanocellulose 3D scaffolds. a) Metal‐ion‐crosslinked nanocellulose hydrogels. b) Confocal laser scanning microscopy images of C3H10T1/2 cells on Ca^2+^‐ and Fe^3+^‐crosslinked nanocellulose hydrogels; i) unmodified Ca^2+^ nanocellulose hydrogel, ii) Ca^2+^ nanocellulose hydrogel with physically adsorbed fibronectin, iii) Ca^2+^ nanocellulose hydrogel covalently attached fibronectin, iv) unmodified Fe^3+^ nanocellulose hydrogel, v) Fe^3+^ nanocellulose hydrogel with physically adsorbed fibronectin, vi) Fe^3+^ nanocellulose hydrogel with covalently attached fibronectin (scale bars: 200 µm). a,b) Adapted with permission.^[^ ^175^ ^]^ Copyright 2014, American Chemical Society. c) Schematic illustration of the composite cellulose‐based cryogel preparation, and photographs of CNF‐Bio showing light weight and high strength. c,d) X‐ray microtomography images of CNF and CNF‐Bio, respectively. e) Force–strain profiles obtained after the uniaxial compression of CNF and CNF‐Bio. f) In vivo evaluation of bone marrow formation performance of cryogels (i) Photographs of the procedure followed in the rat calvarial assay. ii–vi) Representative µCT images of rat calvarial after 56 d of implantation. 3D reconstruction (top row) and 2D slices inside the 3D image (bottom row). ii,v) Control, iii,vi) CNF, and iv,vii) CNF‐Bio. c–f) Adapted with permission.^[^ ^306^ ^]^ Copyright 2019, Royal Society of Chemistry.

Zander et al. studied the C3H10T1/2 fibroblast cell culture in Ca^2+^ and Fe^3+^ metal ion crosslinked carboxylated CNF hydrogels (**Figure** **11**a).^[^ ^175^ ^]^ The CNFs were contained either covalently attached or through physically adsorbed fibronectin onto the nanofiber surface to improve cell adhesion. The metal chelation induced crosslinking enhanced the storage moduli to 3.4 and 32 kPa, respectively for Ca^2+^ and Fe^3+^ crosslinked CNFs. Native CNF hydrogels due to their nanosized pores showed little cellular infiltration. The Ca^2+^ crosslinked with hydrogel without any protein attachment was highly unfavorable for cell attachment and spreading, showing predominantly round shape indicating little or no contact with the gel surface. Cell attachment was similar in Ca^2+^ crosslinked hydrogel with physically adsorbed fibronectin. However, cell spreading and deviation from round morphology was observed. The behavior was altered when the protein was covalently attached. Fe^3+^ crosslinked hydrogels showed better cell spreading than that of Ca^2+^ crosslinked hydrogels and the cell attachment improved with protein containing CNF hydrogels. Cellular coverage increased with protein attachment to the surface. The Ca^2+^‐crosslinked gels with covalently attached protein showed the highest cell spreading (Figure 11b), whereas Fe^3+^ gels showed the highest cell spreading with physically adsorbed protein. Therefore, the attachment of biomolecular adhesive sites improves the cell attachment and cell spreading, especially due to the high concentration of arginine‐glycine‐aspartic acid (RGD) residues. Based on an ELISA assay, it was concluded that Fe^3+^‐crosslinked hydrogels contain less biologically active protein compared to that of Ca^2+^, which explains lower cell attachment and spreading.

Aiming at surgical thread applications, CNF hydrogels were used to prepare threads with a diameter of 0.1 and 0.2 mm using 3D printing for biomedical applications.^[^ ^304^ ^]^ To improve the stability and mechanical properties under wet conditions, the threads were covalently crosslinked (CNF‐X) using glutaraldehyde, which was carefully removed at the end. In their dried state, both CNF and CNF‐X threads showed similar mechanical properties, with a tensile strength of 275 MPa, an elastic modulus of 13 GPa, and a strain at break of 9.5%. Only CNF‐X threads were robust under wet conditions as the unmodified CNF threads showed poor mechanical properties upon wetting. The CNF‐X threads soaked in water were able to retain up to 40% of the dry‐state tensile strength of CNF, resulting in a wet strength close to that of the strongest human tendons. The threads remained stable under cell culture conditions and human adipose stem cells (hASCs) grew more homogeneously along the surface of the CNF‐X threads, whereas the cells attached to CNF were observed to be round and small. The hASCs on CNF‐X displayed an elongated fibroblast‐like shape, characteristic of mesenchymal cells. The CNF‐X coated with ECM promoting laminin also showed similar behavior. CNF‐X threads maintain hASCs undifferentiated profile and functionality. Cells on decorated CNF‐X multifilament threads remain attached after suturing.

The mechanical properties can also be improved using 3D printed biofunctionalized hybrid gel scaffolds. A hybrid TEMPO‐CNF‐alginate 3D printed gel system was reported using avidin‐biotin crosslinking.^[^ ^305^ ^]^ Therein, an ultrastable form of avidin, (nChiAvd) was covalently attached to TEMPO‐CNF and its ability to bind biotin with high specificity and extremely high affinity (*K*_d_ ≈ 6 × 10^−16^ m) was exploited to prevent the shape fidelity and improve the mechanical properties of the scaffolds.

Scaffolds for biomedical applications can also be prepared using cryogel. Ferreira et al. reported hybrid cryogel scaffold of CNF–bioactive glass (CNF‐Bio) for bone regeneration (Figure 11c–f).^[^ ^306^ ^]^ The scaffold consisted of 80% bioactive glass and 20% CNF and preserved the intrinsic moldable properties of CNF despite the presence of a large amount of brittle and fragile bioglass. The low density and high strength cryogel resisted compressive loads over 1250 times its weight. The cryogels showed initiation of the plastic regime under uniaxial compression test 3.5 and 7%, respectively for CNF and CNF‐Bio cryogels. Importantly, the compression strength was also increased from 11 kPa for CNF to 24 kPa CNF‐Bio cryogels under dry conditions. When exposed to simulated body fluid (SBF), the cryogels displayed a drastically different behavior in their ability for hydroxyapatite formation and displayed extensive formation of carbonated apatite layers. The cryogels were studied for in vitro and in vivo osteogenic ability. In vivo bone regeneration was studied using the rat calvarial defect assay (Figure 11f).  μCT assay after 56 days confirmed the partial regeneration of the defect in rat implanted with CNF‐Bio cryogel.

### 3.8 Nanocellulose Hydrogels in Ophthalmic Applications

Hydrogels have been used for various ophthalmic applications, including soft contact lenses,^[^ ^307^ ^]^ foldable soft corrective lenses,^[^ ^308^ ^]^ and ocular drug delivery.^[^ ^309^, ^310^ ^]^ Traditionally synthetic polymer‐based hydrogels, such a poly(hydroxyethyl methacrylate) (PHEMA), poly(methyl methacrylate) (PMMA), poly(vinylpyrrolidone) (PVP), PVAA, cellulose acetate (CA), and silicon‐based materials have studied for various ophthalmic applications.^[^ ^311^ ^]^ For ophthalmic applications, several factors, including biocompatibility, optical properties, mechanical stability and oxygen permeability are important. At the same time, the materials should not elicit any toxicity or immunogenic response. Depending on the application the materials should be sufficiently strong enough to be sutured for implanting and able to hold high water content, but still relatively soft but strong enough mechanically. The eye is one of the highly protected organs and rich in various proteins that may deposit on the surface of the device and alter the structure inducing immune response and inflammation. Therefore, the lenses should be resistant to deposit formation. Hydrogels, because of high water content are comfortable to wear and have lubricity, good oxygen permeability, and resistance against protein deposition. However, due to high water content hydrogel‐based soft lenses are mechanically weak. The extensive discussion on ophthalmic applications of the synthetic and biopolymer hydrogels has been reviewed in the literature,^[^ ^312^ ^]^ therefore, here the focus will be on recent efforts using nanocellulose incorporated hydrogels.

**Figure 12:**
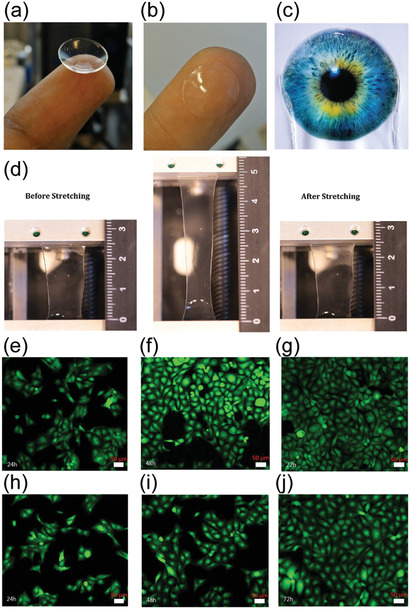
Hybrid nanocellulose hydrogels for ophthalmic applications. a,b) Self‐supporting and transparent contact lens made from 10% PVA and 1% TEMPO oxidized nanocellulose (CNC/CNF) composite hydrogel. a,b) Adapted with permission.^[^ ^314^ ^]^ Copyright 2016, American Chemical Society. c) Image of a transparent hydrogel sheet covering the lower half of the eye (printed image). Reproduced with permission.^[^ ^315^ ^]^ Copyright 2017, Royal Society of Chemistry. d) The elastic behavior of hydrogel in its pristine state, extended state, and relaxed state after stretching. Reproduced with permission.^[^ ^314^ ^]^ Copyright 2016, American Chemical Society. e−g) Confocal microscopy images of GFP‐labeled HCE‐2 cells cultured on CNC‐PVA hydrogel, indicating growth after 24 h (e), 48 h (f), and 72 h (g). h−j) Confocal microscopy images of GFP‐labeled HCE‐2 cells cultured on tissue culture plate (TCP), indicating growth after 24 h (h), 48 h (i), and  72 h (j). e−j) Adapted with permission.^[^ ^313^ ^]^ Copyright 2016, American Chemical Society.

Tummala et al. reported TEMPO‐CNC reinforced PVA hydrogels for ophthalmic applications (**Figure** **12**).^[^ ^313^, ^314^ ^]^ PVA has been used as a viscosity‐enhancing agent in eye‐drops and as an internal lubricant in contact lenses. Previously bacterial cellulose with PVA has been demonstrated for corneal implants but with low optical transparency (75% at 610 nm). The CNC–PVA transparent hydrogel lenses containing 90% of water content showed rubber‐like mechanical properties, soft, elastic, capable of retaining convex shape and displaying 95% transparency in the visible range and moderate UV‐absorption properties (Figure 12a–c). The transparency is attributed to similarities in the refractive indices and interface between CNC and PVA and the CNC width and thickness, that are less than the wavelength of visible light.

The prepared CNC–PVA hydrogel lenses showed a low contact angle of 12^o^, the refractive index of 1.33 is similar to distilled water and high oxygen permeability 66 Å × 10^−11^ Dk, comparable to commercially available contact lenses. Rheologically, CNC–PVA the hydrogel lenses showed high elastic modulus with *G*′ values of 16 kPa. Conventional hydrogel lenses display ductile properties. In contrast, the stress–strain curve of CNC–PVA lenses revealed hyperelastic nature with strength at failure of 0.15 MPa. The gel could be stretched to 300% of its original size (Figure 12d). Suturing experiments showed the PVA–CNC hydrogels were able to tolerate places of 12 interrupted sutures without tearing. Cytocompatibility was tested with corneal epithelial (HCE‐2) cells (Figure 12e–j). The hydrogels promoted the cell growth and cells grew rapidly on the surface of the hydrogel within three days, suggesting a potential corneal implant material. The study was also extended to TEMPO‐CNFs and the hybrid gel showed strain‐induced stiffening behavior.^[^ ^315^ ^]^

### 3.9 Nanocellulose Hydrogels for Fingerprint Detection

**Figure 13:**
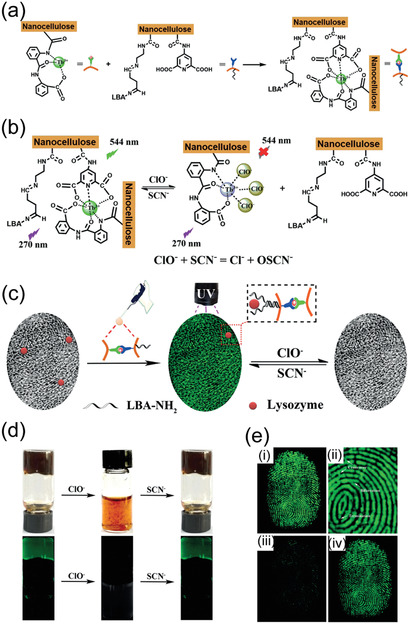
Luminescent nanocellulose hydrogels for latent fingerprint detection. a) Synthesis of the Tb(III) complex‐CNC, LBA–DPY–CNC, and Tb(III)–CNC hydrogel. b) Responsive mechanism upon the addition of ClO^−^ and SCN^−^ anions. c) Concept for latent‐fingerprint detection. d) Photographs of nanocellulose–metal complex in response to ClO^−^ and SCN^−^ anions under (top) visible light and (bottom) UV light. e) Luminescence image of a fingerprint on a microscope slide (i), and corresponding magnified image (ii). iii,iv) Luminescence images of the fingerprint in response to ClO^−^ and SCN^−^ anions. a−e) Adapted with permission.^[^ ^323^ ^]^ Copyright 2018, Wiley‐VCH.

Hydrogels have recently been studied for the application in encryption and decryption process.^[^ ^316^ ^]^ Such applications are useful for rapid detection and encryption of latent fingerprints (LFPs, i.e., fingerprints that are not visible to the naked eye). Fingerprints have been extensively used for biometric identification, access control, medical diagnostics and forensic investigations.^[^ ^317^ ^]^ Historically, materials such as powder dusting (either adhesive polymers with stain or silver, gold, or lead powders), iodine staining, ninhydrin, and various colorants have been used^.[^ ^318^ ^]^ Such detection methods face substantial challenges due to their ineffectiveness under certain circumstances, toxicity and health hazards^.[^ ^319^ ^]^ Therefore, several new materials and methods have recently been developed to detect latent fingerprints,^[^ ^320^ ^]^ and have recently been reviewed.^[^ ^321^ ^]^ One of the goals in LFP detection is to produce an optical contrast between the fingerprint ridges and the surface pattern. Traditionally, various spectroscopy techniques, multimodal deposition, and imaging methods have been used to visualize the LFPs.^[^ ^322^ ^]^ Recently, Hai et al. reported anion responsive luminescent nanocellulose hydrogel for latent fingerprint detection and encryption (**Figure** **13**).^[^ ^323^ ^]^ The hydrogel was prepared by functionalizing carboxylated CNCs using three different pendant units. First, the nanocellulose was functionalized with 2‐(2‐aminobenzamido) benzoic acid unit, which forms a complex with terbium (III) Tb^3+^ (Figure 13a). In another approach, nanocellulose was functionalized with a lysozyme binding aptamer (LBA) and 4‐aminopyridine‐2,6‐dicarboxylic acid (Figure 13b). Upon mixing the two components, the resulting complex led to a gelation and shows green fluorescence when excited at 270 nm. The hydrogels had good photostability, and the fluorescence intensity attenuated by less than 20% when irradiated with an UV lamp for 72 h. Addition of ClO^−^ quenched the fluorescence within 5 s and the fluorescence with recovered within 5 s after adding SCN^−^ ions in the solution and solid state (Figure 13c,d). Moreover, the reversible binding of ClO^−^ also induced sol‐gel transition. The presence of LBA allowed a specific reagent for LFPs, because its folded 3D structure can bind to the lysozyme of fingerprint ridges. When a microscope slide containing a fingerprint was incubated with the hydrogel followed by UV irradiation at 270 nm, bright luminescence on the papillary ridges of LFPs was clearly observed. The luminescence can be erased by treating with ClO^−^ solution, i.e., encryption and recovered by adding SCN^−^, i.e., decryption. The process was repeatable for several cycles.

## 4 Nanocellulose Composite Fibers

**Figure 14:**
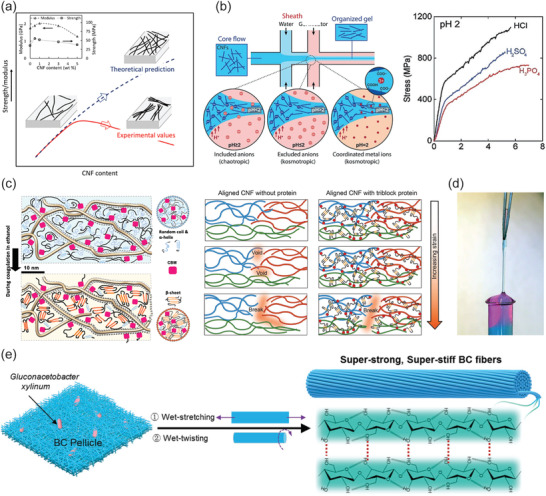
Nanocellulose fibers. a) Schematics illustrating the problem of aggregation to allow high alignment for high strength materials. Reproduced with permission.^[^ ^325^ ^]^ Copyright 2018, American Chemical Society. b) Microfluidic device for flow‐induced alignment and stress–strain curves for high strength CNF fibers. Reproduced with permission.^[^ ^327^ ^]^ Copyright 2019, The Authors, published by Wiley‐VCH. c) Recombinant proteins of a triblock architecture with modified spider silk central block with terminal cellulose affinity modules leading to high toughness in CNF nanocomposites upon alignment. Reproduced with permission.^[^ ^328^ ^]^ Copyright 2019 The Authors, published by AAAS. Reproduced/adapted from ref. [328] © The Authors, some rights reserved; exclusive license American Association for the Advancement of Science. Distributed under a Creative Commons Attribution Non‐Commercial License 4.0 (CC BY‐NC http://creativecommons.org/licenses/by‐nc/4.0/). d) Interpolyelectrolyte fiber spinning of negatively charged TEMPO‐oxidized CNF and cationic polyelectrolyte allowing aqueous spinning of multicomponent fibers. Reproduced with permission.^[^ ^347^ ^]^ Copyright 2017, American Chemical Society. e) Bacterial cellulose spinning from gels followed by twisting leads to high strength fibers. Reproduced with permission.^[^ ^353^ ^]^ Copyright 2017, Wiley‐VCH.

One of the extensively pursued goals of nanocellulose research remains the production of composite fibers of high mechanical properties or combining several functionalities. The first aim is to make materials on a large scale, which would have strengths approaching that of wood nanofibrils, estimated to be in the order of 1 GPa or beyond.^[^ ^324^ ^]^ In terms of scalability and related to processing feasibility, it has been suggested that paper‐like manufacturing processes would be feasible,^[^ ^325^ ^]^ which would, however, lead to sheets of material. However, in terms of being able to capture the strength of nanofibrils, several reports have pointed out that sheet‐like materials lead to entangled fibers, or fibers without orientation in the plane. The lack of orientation and entanglement leads to poor contacts between fibrils and fibrils that are agglomerated in different ways. The weaknesses that result make it difficult to achieve the strength potential of the constituent nanofibers (**Figure** **14**a). On the other hand, reports show that highly aligned nanofibers can be achieved when assembling them into fibers.^[^ ^326^, ^327^, ^328^ ^]^ The results highlight the role of alignment for achieving good fibril contacts and hence properties (Figure 14b).^[^ ^329^ ^]^

Recently, several groups have turned their attention to using sheets or blocks of wood, that are first delignified and then densified.^[^ ^330^, ^331^ ^]^ The advantage is that the native orientation of cellulose fibrils is retained, and sheets with oriented fibers are thus achieved. Compression enhances bonding between fibrils, and strengths of around 350 MPa are achieved in this way.^[^ ^332^, ^333^ ^]^ Taking advantage of the natural orientation in this way is elegant. Much can be learned about the role of alignment and order in nanocellulose materials by this approach. Taking a more commonly attempted approach to breaking down cell walls and then realigning the fibers presents, of course, many challenges, but probably have the advantage of leading to continuous and scalable processes, which offer better prospects for the economy by means of possibilities for automatization of processes. In the dissolution and realigning, one of the main problems is agglomeration and flocculation. The problems become more pronounced as the aspect ratio of the fibrils increases. Since high aspect ratio is a key parameter for making good nanopapers,^[^ ^334^ ^]^ solutions that would decrease aggregation are sought. From a colloid science point of view, the most straight‐forward approach is to increase the electrostatic charge on the surface of the nanofibrils. This will result in the repulsion of like charges, and prevent aggregation and flocculation.^[^ ^335^ ^]^ Here, one has to take care not to exceed the limiting factor for too high a charge density in order to prevent counterion condensation.

The question of surface charge neutralization, and roles during assembly and compression become the next important questions to address. In the cell walls, metal ion complexation is known to play a role in crosslinking, resulting in effects on mechanical stabilities. A well‐known mechanism is that of calcium ions that crosslink the pectin in the middle lamella.^[^ ^336^ ^]^ For nanofibril materials, extensive studies have been conducted to cover multiple parameters such as counterion size and its nature in stabilization and composite properties.^[^ ^337^ ^]^ It was found that both that even only small amounts of counterions had significant effects. Careful balances between colloidal stability, structure formation, and bridging interactions in the final composite must be considered. In a spinning setup, Ca^2+^ ions were found to act as a coagulant for TEMPO oxidized nanofibrils, and affecting also properties of fibers such as moisture uptake and hornification.^[^ ^338^ ^]^ Using another combination, with Fe^3+^ as the counterion and carboxymethylation giving the dispersive modification on the cellulose, a remarkable strength of over 1 GPa was reported for aligned fibers.^[^ ^327^ ^]^

The effects of ions on the properties of hydrogels of nanocellulose become, thus, an important processing step to understand. In the study by Mittal, ions as gel initiator affected carboxymethyl‐nanofibril assembly that was interpreted as a strain stiffening behavior, which is highly remarkable for a nanocellulose material.^[^ ^327^ ^]^ Other reports describe the strong effects of ions, even with celluloses that have not been modified with charged groups for easier processing.^[^ ^339^ ^]^ Overall the conclusion is that even for celluloses with native surfaces ions in general result in higher viscosity and stronger gelling. The degree of compression of the electrical double layer by cations results in aggregation and flocculation. Multivalent cations increase network strength and promote dewatering more than monovalent ones. However, characteristic functions such as shear thinning were not affected.^[^ ^152^ ^]^ The same effects of ions on gel strengths apply for both celluloses that have been surface modified for stability (i.e., TEMPO‐oxidation, carboxymethylation) and celluloses for which the native surface chemistry is unaltered. Although cellulose should be non‐charged, we can expect residual hemicelluloses,^[^ ^340^ ^]^ to contain especially carboxyl groups.^[^ ^341^ ^]^

Biomimetic models suggest that nanocomposites benefit from being designed with polymeric materials to bridge the stiff elements (i.e., the nanocellulose).^[^ ^342^, ^343^ ^]^ This is because polymers can also incorporate bridging effects, hidden lengths, and multiple interactions potentially leading to combinations of strength and toughness. In all‐cellulose composites, the approach is to use cellulose in different forms, both as a bridging matrix, and as the stiff reinforcing components. An approach using lithium/urea dissolved cellulose together with TEMPO oxidized cellulose fibrils demonstrated this approach.^[^ ^344^ ^]^ Other recent developments include the use of silk proteins as the matrix for nanocellulose reinforcements. Native silkworm silk was dissolved in lithium salt, and then regenerated with TEMPO‐modified nanocellulose,^[^ ^345^ ^]^ giving fibers by microfluidic spinning that had both higher stiffness and strength than the natural silk. In another study, recombinantly produced and engineered silk proteins containing cellulose adhesive proteins were combined with cellulose fibrils having nonmodified native surfaces to show toughening and strengthening of aligned nanocellulose fibrils (Figure 14c).^[^ ^328^ ^]^

Overall, we conclude that high performance has been demonstrated by multiple means when it comes to ways of assembling nanocellulose to materials. Much insight has been gained in overall design‐principles and on the special considerations that nanocellulose requires. However, it seems that the aim of combination of high performance, truly green processes, and economic upscaling will continue to pose challenges to researchers in the field.

Another type of nanocellulose fiber formation relates to interpolyelectrolyte fiber spinning (Figure 14d). Two benefits are clear, to spin from water‐based medium and to combine several functionalities. The concept is well established in the field of molecular polymer science.^[^ ^346^ ^]^ To adopt to colloidal level nanocelluloses, anionic TEMPO‐oxidized CNF with a cationic polymer poly(diallyldimethylammonium chloride) allows water‐based spinning of fibers with the mechanical strength not far from the pure CNF.^[^ ^347^ ^]^ Interestingly, additional functionalities dealing with crimping could be achieved. The concept allows additional functional components within the fiber. The approach was also shown by using cationic chitosan and CNF.^[^ ^348^ ^]^ Importantly, chitosan–CNF composite fiber was shown potential for wound healing.^[^ ^349^ ^]^ We also emphasize the postprocessing option to achieve high strength by fiber twisting, inspired by the DNA supercoiling in chromatins.^[^ ^350^ ^]^ Such approaches have extensively been pursued by Baughman′s lab for carbon fibers and fiber actuators.^[^ ^351^ ^]^ Recently, multifunctional fibers have been achieved with tunable mechanical and electrical properties.^[^ ^352^ ^]^ Related to nanocelluloses, twisting of bacterial cellulose macrofibers drawn from the pellicles yield very high tensile strength 826 MPa and Young's modulus of 65.7 GPa.^[^ ^353^ ^]^ We foresee the promising possibilities based on this concept (Figure 14e)

Finally, we shortly point out that beyond the longer CNF and bacterial cellulose nanofibers, also the shorter CNC nanorods offer options for functional fiber spinning upon combining with a polymeric matrix phase. Nanocomposite fibers consisting of CNC and PVA spun from DMSO/water mixtures followed by coagulation and hot‐drawing allow remarkable strength and stiffness reaching 880 MPa and 29.9 GPa.^[^ ^354^ ^]^ On the other hand, flexible transparent fibers with a large strain of 35% and ultimate strength of ca. 200 MPa can be spun from water as a benign medium.^[^ ^355^ ^]^ Therefore the CNC nanocomposite fibers allow highly tunable functional and processing properties.

## 5 Nanocellulose toward Efficient Photosynthetic Cell Factories

Only recently a glimpse into the untapped power of water responsiveness of cellulose‐based assemblies, especially assemblies based on CNFs and CNCs have been revealed. Biological systems often exist in aqueous media, and it is well known that many of their functions depend on interactions with water in liquid and vaporous forms. Upon isolation of nanocelluloses, be it CNFs, CNCs, or microfibrillated cellulose, their high hydroxyl group density and consequential hygroscopic nature is magnified at nanoscale. More importantly, 2D and 3D nanocellulosic assemblies display water transport properties (capillary forces, permeability, and diffusion) which define the actions and the functionality of plant cell wall. In the light of the new evidence on high vapor uptake and binding of water molecules as well as water conveying properties, it can be argued that the unique combination of large surface area and high hygroscopicity is a chief asset when considering future applications operating in aqueous environments.

A fresh approach of utilizing cellulose–water interactions is the introduction of cellulosic materials as biocompatible scaffolds for photosynthetic cell factories (PCFs). Photosynthesis is a unique natural process that harvests solar energy and converts it to chemical energy to support all life forms on Earth. Photosynthetic living cells (microalgae and cyanobacteria) can host novel synthetic production pathways that allows exploitation of photosynthetic microorganisms as microbial cell factories catalyzing the entire process of production targeted solar fuels and chemicals from CO_2_. There are dozens of proofs‐of‐concept for production of diversity of fuels and chemicals using algae and cyanobacteria as hosts.^[^ ^356^, ^357^ ^]^ Today PCF technologies are mainly based on suspension culturing of cells in various configurations of bioreactors, and such systems do suffer from self‐shading leading to a low light utilization efficiency, low volumetric cell density, loss of energy due to excessive mixing, competing metabolic pathways, and intensive biomass accumulation instead of production of desired chemicals, as well as high water consumption.^[^ ^358^ ^]^

The controlled whole‐cell immobilization is a proved strategy to improve the cell factory efficiency and bring solutions to the above‐mentioned challenges. A recent review,^[^ ^359^ ^]^ collects the state‐of‐the art microalgae and cyanobacteria immobilization strategies addressing the clear benefits of the thin‐layer immobilization approach. Briefly, controlled immobilization is defined as a method that applies chemical, physical, or mechanical forces to integrate living cells to a mechanical scaffold. Essentially, key factors dictating the efficiency and feasibility of the controlled thin‐layer cell immobilization strategy are: i) the improvement in the light utilization efficiency of the cells (increased light‐to‐product conversion)^[^ ^360^ ^]^; ii) the restriction of cell division as the matrix limits the cell growth and directs the energy towards the desired chemicals; iii) high volumetric cell density; and iv) significantly prolonged biocatalytic production times.^[^ ^361^, ^362^ ^]^

Extensive literature on microalgae immobilization strategies exists involving vast selection of matrix materials varying from biobased polymeric matrices (e.g., alginate, carrageenan, agar, chitin) to synthetic counterparts (e.g., PVA, polyethylenimine, silica sol–gel), alginate being the most popular alternative.^[^ ^359^, ^361^, ^362^, ^363^, ^364^, ^365^ ^]^ Hardly any entries where cellulose‐based immobilization substrates for microalgae have been utilized can be found. *Trentepholia aurea* forming biofilms on filter paper,^[^ ^366^ ^]^ and *Phromidium laminosum* immobilized in cellulose fibers^[^ ^367^ ^]^ have been used for nitrogen and/or phosphorus removal from waste waters. Balusamy et al.^[^ ^368^ ^]^ discusses the encapsulation of virus in cellulose diacetate–PEO matrix using electrospinning but the approach does not involve microalgae. A thorough review effort on microalgae immobilization^[^ ^359^ ^]^ did not yet deal with the subject of cellulose as a matrix material for photosynthetic cell factories, not to mention the exploitation of highly hygroscopic nanocellulosic networks. The first attempt to reveal the potential of nanocellulose in the context of microalgae immobilization was recently introduced.^[^ ^369^ ^]^

Nanocellulosic networks provide multiple benefits over the conventional immobilization matrices to confine living cells. An ideal matrix material for PCF: i) does not hamper vital cellular functions by facilitating optimal distribution of light, micronutrients, water, CO_2_; ii) efficiently releases of the target chemicals into the surrounding medium or gas phase, and iii) restricts biomass accumulation. These are the key factors for converting thin‐layer microbial films into high turn‐over direct photobiocatalysts. Conventional gel entrapment using, e.g., alginate has limitations with respect to mechanical stability and diffusion of water and gases due to low porosity. This causes accumulation of photosynthetically evolved O_2_ inside the matrix inducing photoinhibition and oxidative stress on cells. Most importantly the system does not allow big molecules such as, e.g., sesquiterpenes to pass through the matrix and be released out, thus limiting application of biofilm technology in microbial cell factories.

**Figure 15:**
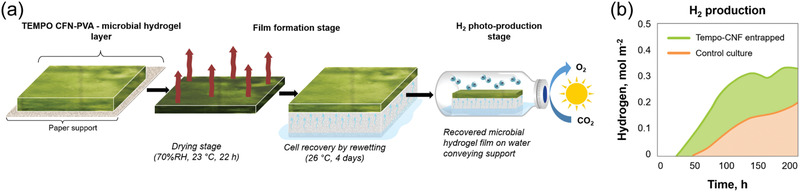
a) Schematic illustration of the concept where cyanobacteria is embedded in TEMPO CNF hydrogel which is crosslinked with PVA during drying to enhance the matrix wet strength. Cells are recovered by rewetting the matrix by moisture which is conveyed through a porous support. Finally, the solidified system is exposed to H_2_ photoproduction conditions and the recovered microbial/CNF thin‐layer was able to continue H_2_ production. b) H_2_ production of cyanobacteria cells after drying and rewetting sequences. Note, that the alginate matrix gave similar results compared with PVA crosslinked TEMPO CNF matrix. Control culture (cells without any matrix) significantly suffered from the drying. a,b) Adapted under the terms of the CC‐BY Creative Commons Attribution 3.0 Unported License (https://creativecommons.org/licenses/by/3.0).^[^ ^369^ ^]^ Copyright 2018, Royal Society of Chemistry.

It is well‐established that 3D CNF hydrogels are translucent or fully transparent depending on the dimensions and size distribution of individual fibrils, alike the 2D film structures formed upon drying. Along with their peculiar optical performance ensuring the sufficient light‐utilization efficiency for the cells, the nanoscaled fibrillar network provides high porosity coupled with high surface area promoting the diffusion of gases, nutrients, and water. The very same features also assure the improved mechanical performance, i.e., high gel strength and tailorable wet strength via chemical crosslinking yielding good matrix durability also in aqueous environments. Last but not least, numerous surface functionalization options for CNF and CNF matrices exists and the matrix porosity can be tuned—attributes that facilitate controllable cell attachment and enable routes to optimize influx and efflux of gases and water. A proof‐of‐concept was demonstrated,^[^ ^369^ ^]^ taking into account all the above‐mentioned characteristics, how cyanobacterial and green algal cells can be embedded in TEMPO CNF hydrogels or in solid films simultaneously maintaining their photosynthetic activity and H_2_ photoproduction capacity. A few crucial findings were generated: i) Various TEMPO CNF matrix materials (pristine hydrogels, Ca^2+^ crosslinked hydrogels and PVA‐crosslinked solid films) were all biocompatible matrices for the studied photosynthetic microorganisms (filamentous cyanobacterium *Anabaena* sp. PCC7120 and green alga *Chlamydomonas reinhardtii*). ii) The H_2_ production activity of the CNF immobilized cyanobacterial cells was similar to alginate matrix. iii) Green algal cells which were entrapped in Ca^2+^ crosslinked TEMPO CNF hydrogel yielded higher amounts of H_2_ after 300 h than cells which were entrapped inside the similarly crosslinked alginate matrix. Here the porous nature of TEMPO CNF network was speculated to play a key role which probably promoted the efflux of H_2_. iv) When embedded in PVA crosslinked TEMPO CNF matrix, cyanobacterial cells fully recovered their photosynthetic activity and they were able to produce H_2_ after drying and rewetting cycles. This approach is depicted in **Figure** **15**a and the H_2_ production after rewetting of the matrix is shown in Figure 15b. In this case, the behavior is assumed to be rooted in high moisture sensitivity and specific water interactions of TEMPO CNF network. TEMPO CNF film is able to retain and bind a relatively high amount of water.^[^ ^370^ ^]^ At the relative humidity levels higher than 60% clustering of water molecules on the surface of the TEMPO CNF takes place and it seems that moisture protects cells during drying.

However, the reported system is still categorized as a passive system where the cells are randomly distributed in the nanocellulose gel and they are not specifically attached on the CNF surface. The next step is to tailor the 3D “leaf” architecture toward more active and responsive direction. The system enables not only the optimization of light harvesting and chemical production and secretion out; it also puts forward a completely new materials template where gels and solid films with controlled pore size, water content, diffusion control and optical properties can be manufactured on spot. This is completely different approach from just randomly mixing cells in a hydrogel template. The system will widen opportunities further for the development of the biohybrid systems, where photosynthetic microbes function as biological photoelectrochemical cells (BES or BPVs) harvesting solar energy and generating electrical current or acting as a component of a “bionic leaf.”^[^ ^371^ ^]^

## 6 Conclusion

We have emphasized some of the recently developed functionalities that we consider particularly promising in relation to nanocelluloses. In general, the different forms of nanocelluloses have already attracted considerable and growing attention in the context of sustainable nanotechnology. Therein, different functionalities and applications have been foreseen by exploiting the rod‐like character of CNCs, or alternatively the more flexible and longer character of CNFs and bacterial cellulose. For example, extensive efforts have been invested to reinforce nanocomposites where considerable progress has been witnessed. Other widely explored areas include nanocellulose films for packaging or for supporting soft devices, and using nanocellulose as viscosity modifiers. Furthermore, structural colors based on CNC assembly have received ample attention, even to the point of artistic design.

Beyond the already‐reviewed applications, herein we have focused on fundamental advances in cellulose science and on applications where we see significant potential. Fundamental discoveries on cellulose structure, its chemical reactivity, and its interactions with water have been unveiled on an unprecedented scale during the past two decades. All these aspects are intrinsically linked to the applications reviewed in here: how the more precise knowledge on chemical modification of nanocellulose can provide subtle tuning capabilities to its interactions and assemblies, relevant in hydrogel properties or regeneration phenomena upon fiber preparation, among others. Here, the colloidal character of nanocellulose adds another dimension to the package. Furthermore, virtually all self‐assembly—be it in hydrogel crosslinking, film formation, viscosity modification, or fiber regeneration—is governed ultimately by cellulose/water interactions.

When the aforementioned fundamentals are projected into hydrogels by tuning the stiffness of the hydrogel skeletons, architectures, and types and densities of crosslinks, one can achieve either extreme softness or stiffness, strain induced stiffening or rigidification, toughening and fracture energy dissipation, phase transitions, biological adhesion, and material or electric transport properties. Especially the hybrid gels allow for various stimulus‐responses, shape‐memory effects, self‐healings, gluing, biological applications, and fingerprint detection. As a potentially emerging promising new application, we review nanocellulose as a platform for photosynthetic microbial cell factories. In summary, we hope that the present review encourages toward so far less emphasized new research directions using nanocelluloses, exploiting their in‐depth structural tunings. This could be relevant for the next generation of smart materials and biological functions.

## Conflict of Interest

The authors declare no conflict of interest.
